# Contaminants of emerging concerns (CECs) in a municipal wastewater treatment plant in Indonesia

**DOI:** 10.1007/s11356-022-23567-8

**Published:** 2022-10-22

**Authors:** Maryani Paramita Astuti, Suprihanto Notodarmojo, Cindy Rianti Priadi, Lokesh P. Padhye

**Affiliations:** 1 Department of Civil and Environmental Engineering, The University of Auckland, Auckland, New Zealand; 2 Environmental Engineering Study Program, Faculty of Engineering, President University, Cikarang, Indonesia; 3 Environmental Engineering Department, Faculty of Civil and Environmental Engineering, Bandung Institute of Technology (ITB), Bandung, Indonesia; 4 Environmental Engineering Study Program, Civil Engineering Department, Engineering Faculty, University of Indonesia (UI), Depok, Indonesia

**Keywords:** Contaminants of emerging concerns (CECs), Occurrence and fate, Wastewater treatment plants, Waste stabilization pond (WSP), Indonesia

## Abstract

This study provides the first set of quantitative data on the occurrence and fate of a wide range of contaminants of emerging concerns (CECs) in Indonesia’s largest wastewater treatment plant (WWTP). The WWTP employs waste stabilization ponds (WSPs) as the secondary treatment before discharging the effluent to the Citarum River. Fourteen out of twenty-two monitored CECs were detected in the wastewater influent, and seven were present in the effluent, with a total concentration of 29.8 ± 0.4 µg/L and 0.5 ± 0.0 µg/L, respectively. The occurrence of the CECs in this study was found to be well correlated with their possible use and known detection in surface waters in Indonesia. Caffeine (CAF) at 12.2 ± 0.1 µg/L, acetaminophen (ACT) at 9.1 ± 0.1 µg/L, *N*,*N*-diethyl-*m*-toluamide (DEET) at 5.0 ± 0.1 µg/L, ibuprofen (IBU) at 2.3 ± 0.0 µg/L, and triclosan (TCS) at 470 ± 64 ng/L were discovered as the five most prevalent CECs, followed by bisphenol A (BPA), trimethoprim (TMP), Tris(2-chloroethyl) phosphate (TCEP), sulfamethazine (SMZ), carbamazepine (CBZ), fluoxetine (FLX), benzotriazole (BTA), sulfamethoxazole (SMX), and metformin (METF). Biodegradable CECs (SMX, SMZ, ACT, IBU, TCS, BPA, CAF, DEET, and TMP) were efficiently removed (83–100%) by the WSP. In contrast, recalcitrant CECs achieved poor removal efficiencies (e.g., FLX at 24%), and for others, treatment processes even resulted in elevated concentrations in the effluent (CBZ by 85%, TCEP by 149%, and BTA by 92%). The CECs’ influent concentrations were determined to pose a moderate aquatic cumulative risk, while no such risk was associated with their effluent concentrations. The study demonstrates the importance of conventional WWTPs in reducing the concentrations of CECs to minimize their aquatic contamination risk. The findings are relevant for countries, such as Indonesia, with limited resources for advanced centralized wastewater treatments, and which are exploring the efficacy of centralized WSP against the existing decentralized treatments.

**Supplementary Information:**

The online version contains supplementary material available at. 10.1007/s11356-022-23567-8.

## Introduction

**Fig. 1:**
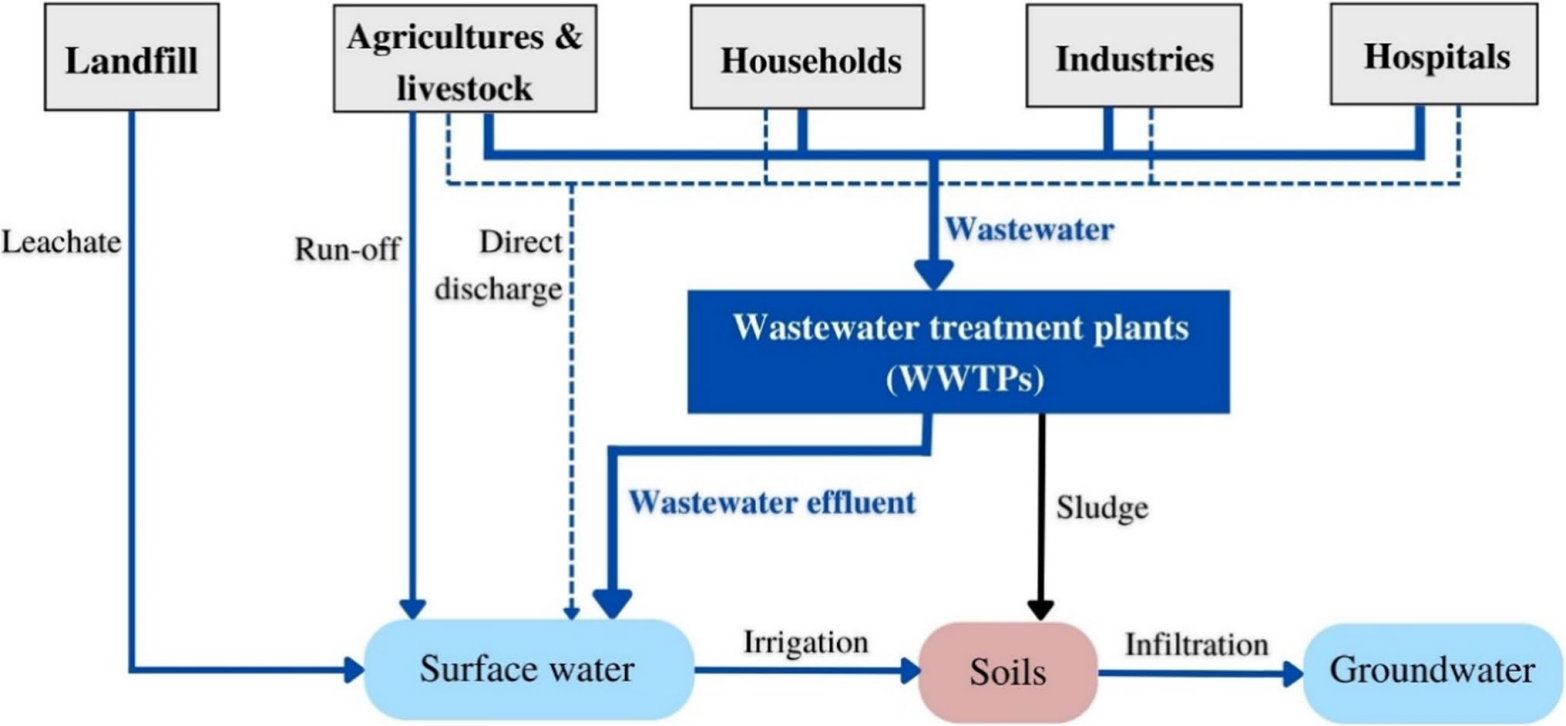
Sources and pathways of CECs in the environment

The contaminants of emerging concerns (CECs) cover a wide range of anthropogenic compounds, i.e., pharmaceuticals and personal care products (PPCPs), pesticides and herbicides, additives, steroid hormones, disinfection byproducts, etc. CECs are water pollutants due to their known or potential hazards to human health and/or the aquatic environment (Rodriguez-Narvaez et al. 2017; Tran et al. 2018). Some of these CECs are known for their chronic toxicity, carcinogenicity, endocrine disruption capacity, and increasing antibiotic resistance (Zad et al. 2018). CECs enter the environment through different sources and pathways as shown in Fig. 1.

Globally, many studies confirm the presence of CECs in wastewater (Saidulu et al. 2021; Starling et al. 2019). Due to the incomplete removal of CECs by conventional wastewater treatment plants (WWTPs), wastewater effluent has been identified as one of the primary sources of CECs in the environment (Rathi et al. 2021). Their presence in wastewater effluent also creates additional challenges for wastewater reuse practices (Parida et al. 2021), as intentional or unintentional indirect reuse results in some CECs being detected in drinking waters (Padhye et al. 2014).

Considering the probable hazards of CECs on the environment and human health, understanding their occurrence and fate in WWTPs is essential for risk assessment and planning necessary mitigation actions (Couto et al. 2019). Previous studies discovered considerable variations in the types and concentrations of CECs detected in WWTPs across different cities and countries, notably influenced by their chemical consumption patterns and socioeconomic profiles (Khan et al. 2020; Saidulu et al. 2021). CECs’ removal efficiencies by WWTPs also vary depending on their properties, climatic conditions, treatment types, and the WWTPs’ operational parameters (Das et al. 2017; Rodriguez-Narvaez et al. 2017).

Despite numerous studies available in the literature on the occurrence and fate of CECs in WWTPs, the majority of those are concentrated in certain geographical areas (Parida et al. 2021; Tran et al. 2018). The data regarding the identification and quantification of CECs in developing countries is still scarce (Khan et al. 2020). In Asia, studies in this field were conducted mainly in Northeast Asia, leaving a significant knowledge gap in Southeast Asia (Tran and Gin 2017). In the region, tons of residual antibiotics are estimated to be discharged into the environment annually without proper documentation regarding the level of antibiotic residue contained in hospital, industrial, and municipal wastewater (Lundborg and Tamhankar 2017). Additionally, the control over the distribution and usage of pharmaceuticals in developing countries is inadequate (Chereau et al. 2017). In Indonesia, for example, unnecessary prescription of antibiotics and antimicrobials and their unprescribed use are commonplace practices (Parathon et al. 2017). These circumstances result in high pharmaceutical consumption rates, including those of antibiotics, in the country (Limato et al. 2022; Parathon et al. 2017). The pharmaceuticals’ consumption rates in highly populated countries are expected to rise rapidly due to their high population (Patel et al. 2019).

Until 2020, there was only one study reporting the occurrence of CECs in the natural or engineered aquatic systems in Indonesia (Menon et al. 2020). That study identified a group of antibiotics in a river, the Ciliwung River, impacted heavily by sewage (Shimizu et al. 2013). Although a recent study detected a high level of acetaminophen (400–600 ng/L) in Jakarta Bay (Koagouw et al. 2021), there is no information on the levels of CECs in WWTPs of Indonesia, which are attributed as the primary sources of detected CECs in those two studies.

This work presents the first study on the occurrence and fate of CECs in a municipal WWTP in Indonesia, the world’s fourth most populous country, employing waste stabilization pond (WSP) system. The WWTP is located in one of the megacities of Indonesia. Apart from investigating CECs’ occurrence concentrations and removal efficiencies in a WWTP, the study has critically reviewed and discussed (1) the possible sources of the detected CECs and their consumption nature in the city and the country, (2) the comparison of occurrence and removal of the CECs from this study with their reported concentrations in surface waters in Indonesia as well as with those reported in other countries and other WWTPs employing WSP, and (3) the removal mechanism of the detected CECs by the WWTP. This study would fill a significant knowledge gap on CECs in Indonesia and contribute to global knowledge development, as WSP is one of the most prevalent treatments practiced worldwide, especially in developing countries.

## Material and methods

### Selection of CECs

Twenty-two compounds from different classes of CECs were selected: trimethoprim (TMP), clarithromycin (CLTR), sulfamethoxazole (SMX), and sulfamethazine (SMZ) of antibiotics; acetaminophen (ACT), naproxen (NPX), diclofenac (DFC), and ibuprofen (IBU) of non-steroidal anti-inflammatory drugs (NSAIDs); triclosan (TCS) of antimicrobials; atenolol (ATL) and metoprolol (MPL) of beta-blockers; carbamazepine (CBZ) and fluoxetine (FLX) of anticonvulsants/antidepressants; benzotriazole (BTA) of additives; bisphenol A (BPA) of plasticizers; caffeine (CAF) of stimulants; *N,N*-diethyl-*m*-toluamide (DEET) of insect repellents; atrazine (ATZ) of herbicides; Tris(2-chloroethyl) phosphate (TCEP) of flame retardants; metformin (METF) of antidiabetics; estrone (E1) of steroid hormones; and *N*-nitrosodimethylamine (NDMA) of disinfection byproducts (DBPs). The CECs were selected based on at least one of the following considerations: (i) extensively used worldwide (Loos et al. 2013; Xu et al. 2021), (ii) frequent detection in wastewater in Asia and across different countries (Parida et al. 2021; Tran et al. 2018), (iii) environmental and human health risk associated with extremely low concentrations (Huang et al. 2020; Menon et al. 2020), and (iv) low removal efficiencies by conventional WWTPs (Rathi et al. 2021; Starling et al. 2019). The selected CECs and their chemical structures are listed in Table S1 of Supporting Material.

### Chemicals and consumables

The CECs’ reference standards (≥ 95% purity) were obtained from Sigma-Aldrich (New Zealand). Dichloromethane (DCM) Optima American Chemical Society (ACS) grade, acetonitrile Optima liquid chromatography/mass spectrometry (LC/MS) grade, and methanol Optima LC/MS grade were obtained from Fisher Scientific (New Zealand). For preparation of samples in Indonesia, DCM LC grade and methanol LC/MS grade were purchased from Merck (Indonesia). All other chemicals used in this study were of analytical grade. Millipore 0.7-µm glass fiber filters were purchased from Sigma-Aldrich (New Zealand). Oasis HLB 6 cc cartridges (with 500 mg of sorbent) were procured from Alphatech (New Zealand). Supelco Coconut Charcoal SPE cartridges (2 g, 6 cc) were obtained from Sigma-Aldrich (New Zealand). Stock solutions of 1000 mg/L concentration of each CEC were prepared in methanol and stored in an amber borosilicate bottle at − 18 °C prior to use. Milli-Q water produced by Milli-Q® Direct 8/16 System from Millipore SAS was used in all experiments (resistivity 18.2 MΩ·cm). All glassware was washed with diluted decon 90 and tap water, then rinsed twice with Milli-Q water and dried before use.

### Characteristics of the studied areas and the wastewater treatment plant

**Fig. 2:**
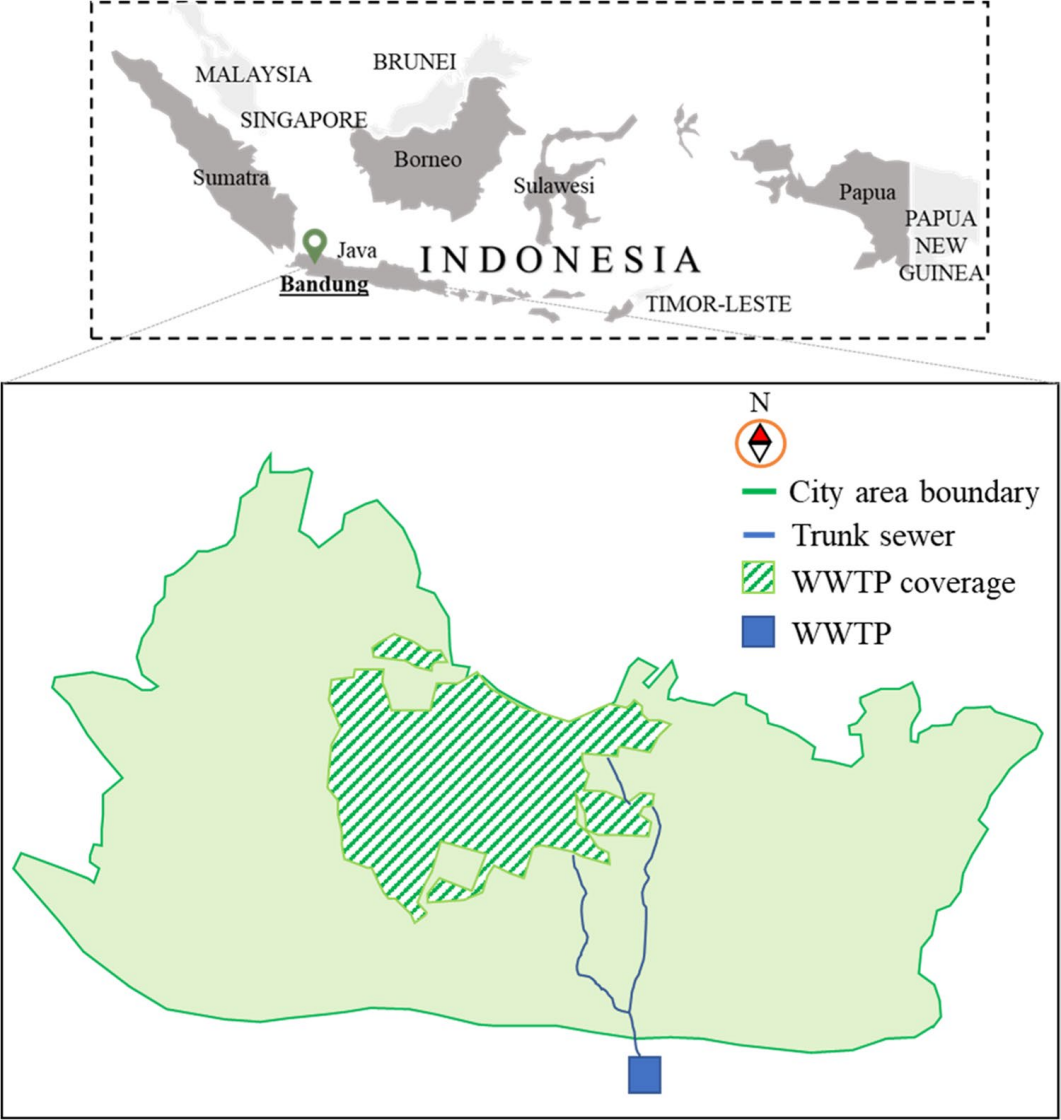
Location of Bandung City, the WWTP, and its coverage area

The centralized wastewater treatment in Indonesia, the largest and most populous country in Southeast Asia, is only available in twelve cities, covering less than 5% of the total population (World Bank 2013). The majority of the population relies on basic septic tanks, while the remaining directly discharges their wastewater into water bodies without any treatment (World Bank 2013). The studied WWTP is located in the city of Bandung, the capital of the West Java Province. It is the largest WWTP in Indonesia and serves the eastern part of the city, around 43% of the population, at a 40,000 m^3^/day operational capacity, although its total capacity is listed at 80,000 m^3^/day (Purwanto et al. 2017; SPKP 2018). The limited sewerage infrastructure in the city and the household’s unwillingness to connect to the centralized wastewater system have resulted in the low utilization of the WWTP (World Bank 2013). The location of Bandung City, the WWTP, and its service coverage area are shown in Fig. 2.

**Fig. 3:**
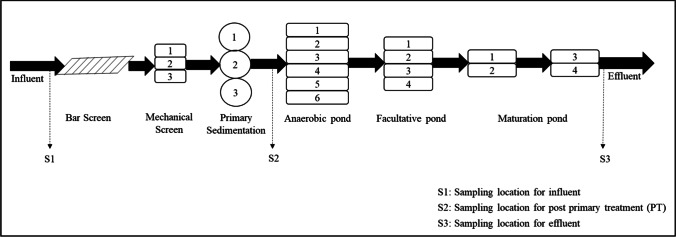
The schematic diagram of the WWTP and sampling locations

The WWTP has primary treatment, comprising of bar screens, mechanical screens, and primary sedimentations, with a hydraulic retention time of 1.5–2 h. The secondary treatment of WSP consists of anaerobic ponds, facultative ponds, and maturation ponds. Figure 3 presents the schematic diagram of the WWTP. The design and operational parameters of the WSP system are listed in Table S2. The wastewater effluent is discharged to the Citarum River, the largest river in the West Java Province, which flows to the Saguling Dam. The Citarum River is known as one of the most heavily polluted rivers in Indonesia and is listed as one of the most polluted rivers worldwide (Hairan et al. 2021). Considering the increasing water demand in Bandung City, the Saguling Dam is projected to be a promising water source for the city (Afiatun et al. 2018). The WWTP itself has a long-term plan to reuse the wastewater effluent for potable water supply (Purwanto et al. 2017). Hence, the results of this study have added importance for future planning.

### Sample collection

The 24-h composite samples were collected at three sampling locations: (1) S1 at the inlet of the WWTP (influent), (2) S2 at the outlet of primary treatment (PT), and (3) S3 at the outlet of the WWTP (effluent), as illustrated in Fig. 3. The samples were collected hourly using vertical Van Dorn bottle samplers (7.00 AM to 6.00 AM) on 18 June 2019 during dry weather. The 200-mL hourly collected samples were placed in borosilicate glass bottles covered with aluminum foil and were kept at 4 °C in the dark throughout the sampling period. The twenty-four samples from each sampling point were then mixed to form a composite sample with a total volume of 4.8 L. The samples were transported to the laboratory in cooler boxes filled with icepack immediately upon the sampling (Tran and Gin 2017).

### Sample preparation and solid-phase extraction

Immediately upon arrival in the laboratory, samples were acidified by adding hydrochloric acid and were stored at 4 °C (EPA 2007). Within 5 days of sampling, acidified samples were filtered using 0.7-µm glass fiber filters. On the following day, solid-phase extraction (SPE) was performed for unspiked and spiked samples, with a 500 mL volume for each of the duplicates. For spiked samples, 500 mL of filtrate samples was spiked with 2 µg/L of target CECs prior to SPE. The SPE was conducted for the target CECs except for NDMA (twenty-one CECs), employing Oasis HLB cartridges with the protocol mentioned in our earlier published work (Astuti et al. 2022). For NDMA, the SPE was conducted using Supelco Coconut Charcoal SPE cartridges according to SPE procedures stated in our previous published work (Astuti et al. 2021). However, due to difficulty procuring new NDMA stock, the compound was not spiked during the SPE; hence, the analysis for NDMA was performed only to check its presence or absence in the sample (detect/non-detect).

The SPE was performed in Indonesia up to sample loading and drying of cartridges steps. The dried cartridges were frozen and shipped in cold condition to the University of Auckland (New Zealand) following the procedures specified in similar works (Archer et al. 2018; Shimizu et al. 2013). Directly after arrival at the University of Auckland laboratory, the cartridges were thawed to room temperature and eluted with 10 mL of DCM (for charcoal cartridges) and with 10 mL of methanol (for HLB cartridges). Subsequently, the eluted samples were concentrated to 1 mL with a vacuum concentrator (Savant RVT5105, Thermo Scientific). The concentrated samples were analyzed by gas chromatography mass spectrometry (GC/MS) for NDMA and liquid chromatography-tandem mass spectrometry (LC–MS/MS) for other CECs. Analysis conditions are described in the following section. Shimizu et al. (2013) compared the results of fully conducted SPE procedures on antibiotics in a local laboratory with those using cartridges shipped overseas with the same protocol that was adopted in this study. They reported that the difference in the readings was less than 25%.

### Analytical methods

The analysis of NDMA was performed on Shimadzu GC-2010 GC/MS employing an HP5-5MS-Ui column (29.1 m length × 0.25 µm ID × 0.25 µm thickness) according to our previous work (Astuti et al. 2021). The analysis of the remaining twenty-one CECs was carried out on a Shimadzu 8040 LC–MS/MS. Multiple reaction monitoring (MRM) mode was used to analyze precursor and product ions of each compound employing an Ascentis reverse-phase amide column (10 cm × 2.1 mm, 3 µm). Both positive and negative electrospray ionization (ESI) modes were employed. The analysis of the compounds, except E1, was merged into a single analytical method with a binary gradient consisting of 5 mM of ammonium acetate in Milli-Q water (A) and methanol (B) at a flow rate of 0.2 mL/min. The injection volume was 2 µL. The oven temperature was set at room temperature. The desolvation line temperature and heat block temperatures were kept at 250 °C and 400 °C, respectively. The nitrogen was used as nebulizing gas (3 L/min) and drying gas (15 L/min). The mobile phase program of the method is shown in Table S3. A separate method was developed for E1 analysis employing the same column using 0.4 mM of ammonium fluoride buffer in Milli-Q water (A) and a mixture of acetonitrile and methanol (1:1) (B) at a flow rate of 0.2 mL/min with the total program of 7 min (85% B) as reported in our earlier work (Astuti et al. 2022). The MS acquisition of the CECs analyzed by LC–MS/MS is listed in Table S4.

### Quality assurance and control

The target CECS were identified based on the retention times and the ratio between quantifier and qualifier ions (Kumar et al. 2019; Shimizu et al. 2013). For quantification, the external calibration curves were plotted from a linear regression relationship between concentrations of standards and their peak areas with a range of correlation coefficients (*R*^2^) > 0.99 for all the CECs. All samples were collected and analyzed in duplicates (*n* = 2). The limit of detection (LOD) and limit of quantification (LOQ) were determined at signal-to-noise (S/N) ratios of 3 and 10, respectively (Kumar et al. 2019). The LODs and LOQs for the concentration factors used in the study and CECs’ average recoveries in all three water matrices are listed in Table S5. The LODs and LOQs in this study were in the range of 1 to 109 ng/L and 3 to 360 ng/L, respectively.

### Extraction recoveries

The extraction recoveries were determined by the matrix spike method, which were calculated by subtracting the analyzed concentrations of the unspiked samples from the analyzed concentrations of the spiked samples and dividing that difference by the known concentration of spiked CEC (Kumar et al. 2019). The extraction recoveries used to quantify the compounds in the samples are shown in Table S5. In general, the extraction recoveries were higher for effluent samples than for the influent and PT samples, consistent with the literature (Gusmaroli et al. 2018). The average recoveries for each CEC were in the range of 39–131%. For four CECs (METF, ATL, CLTR, and SMX), the average recoveries were found to be lower than 20%. Hence, the analysis for these four CECs was performed only to check their presence or absence in the sample (detect/non-detect). Low SPE recoveries of these CECs (≤ 20%) in wastewater using the same SPE cartridge have been reported previously (Kumar et al. 2019). Similarly, low SPE recoveries of SMX in wastewater were reported by Zhang et al. (2020) at 24%. The notably poor recoveries (≤ 5%) of metformin from water and wastewater by HLB are attributed to the high polarity of metformin (Kumar et al. 2019). Gros et al. (2006) compared recoveries of pharmaceuticals using Oasis HLB cartridges at neutral and acidic pH. They found the low recoveries of ATL (< 20%) and other neutral and basic compounds in acidified samples, which could be one of the reasons for the low recoveries of ATL (p*K*_a_ 9.6), CLTR (p*K*_a_ 8.4), and SMX (p*K*_a_ 6.16) in our study.

### Wastewater quality characterization

The pH of the samples was measured using an Agilent 3200P pH meter. Total organic carbon (TOC) and total nitrogen (TN) were analyzed using a TOC-L analyzer from Shimadzu. Total phosphorus (TP) was measured according to the Standard Methods 4500-P colorimetric method. Additional parameters, total suspended solids (TSS), biological oxygen demand (BOD), chemical oxygen demand (COD), and ammonia (N-NH_3_), were adopted from the weekly water quality monitoring of the WWTP from January 2019 to June 2019.

## Results and discussions

### Wastewater quality characterization

The characteristics of the wastewater in this study are shown in Table 1. The average pH of influent and effluent samples, 7.6 and 8.4, respectively, indicated that the wastewater had a low share of industrial wastewater (Camacho-Muñoz et al. 2012). The pH was also within the range suitable for biological treatment (6–9) (Wijaya and Soedjono 2018). Typical domestic wastewater is classified into three categories: weak, medium, and strong, based on its characteristics, as listed in Table S6. TSS, BOD, COD, TP, and N-NH_3_ found in the influent indicated that the wastewater was considered “weak”. TSS is one of the important parameters that determine the removal of CECs through adsorption onto solid particles (Camacho-Muñoz et al. 2012). The organic substances and nutrients contained in wastewater are key factors in promoting the biodegradation of CECs (Camacho-Muñoz et al. 2012). The parameters found in the wastewater were also lower than their reported values from other WWTPs employing WSP, as presented in Table 1. The low concentrations of these parameters could be caused by dilution effects from the infiltration of groundwater, stormwater, and irrigation water into the sewerage system (Dirckx et al. 2016; Hijosa-Valsero et al. 2010).

**Table 1:** Wastewater quality parameters and the removal efficiencies from this study and other WWTPs with WSP system

Parameters	This study			Effluent discharge limits in Indonesia^a^	Other WWTPs with WSP system		
	Influent	Effluent	Removal (%)		Influent	Effluent	Removal (%)
pH	7.6	8.4	–	6–9	7.4–8.6^b^	7.7–9.2^b^	–
TSS* (mg/L)	64	24	62.5	30	134–254^b,c^	17–44^b,c^	54–93^b,c^
BOD* (mg/L)	90	27	70	30	125–440^d,e,f^	10–135^d,e,f^	60–82^d,e,f^
COD* (mg/L)	173	90	48	100	298–464^b,f^	40–221^b,f^	38–78^b,f^
TOC (mg/L)	16.8	5.5	67.3	–	21–127^ g,h^	10–46^ g,h^	− 44 to 86^ g,h^
TN (mg/L)	11.5	2.6	77.4	–	172–376^b^	8–222^b^	62–99^b,i^
TP (mg/L)	0.5	0.4	20.8	–	0.8–19^b^	0.5–13^b^	22–99^b,i^
N-NH_3_* (mg/L)	8.4	3.1	63.1	10	12–38^b^	0.5–18^b^	53–96^b^

*Data from the WWTP for January 2019–June 2019.

^a^KLHK (2016).

^b^Camacho-Muñoz et al. (2012).

^c^Matamoros et al. (2016).

^d^Hijosa-Valsero et al. (2010).

^e^Ensink et al. (2007).

^f^Tyagi et al. (2008).

^g^Li et al. (2013).

^h^Sabah et al. (2016).

^i^Hoque et al. (2014).

The removal efficiencies of these parameters in Table 1 indicated that the WWTP had moderate performances (48–77%). COD removal was considerably low (48%), which might be attributed to influent overloading and sludge accumulation, resulting in a short hydraulic retention time (Edokpayi et al. 2021). Considering that the WWTP did not experience overloading when the study was conducted and it had an irregular desludging schedule, the low COD removal could be due to the sludge accumulation in the ponds. It was also found that the wastewater effluent met the Indonesian domestic wastewater discharge limits, as shown in Table 1.

### Occurrence of CECs in the WWTP

**Fig. 4:**
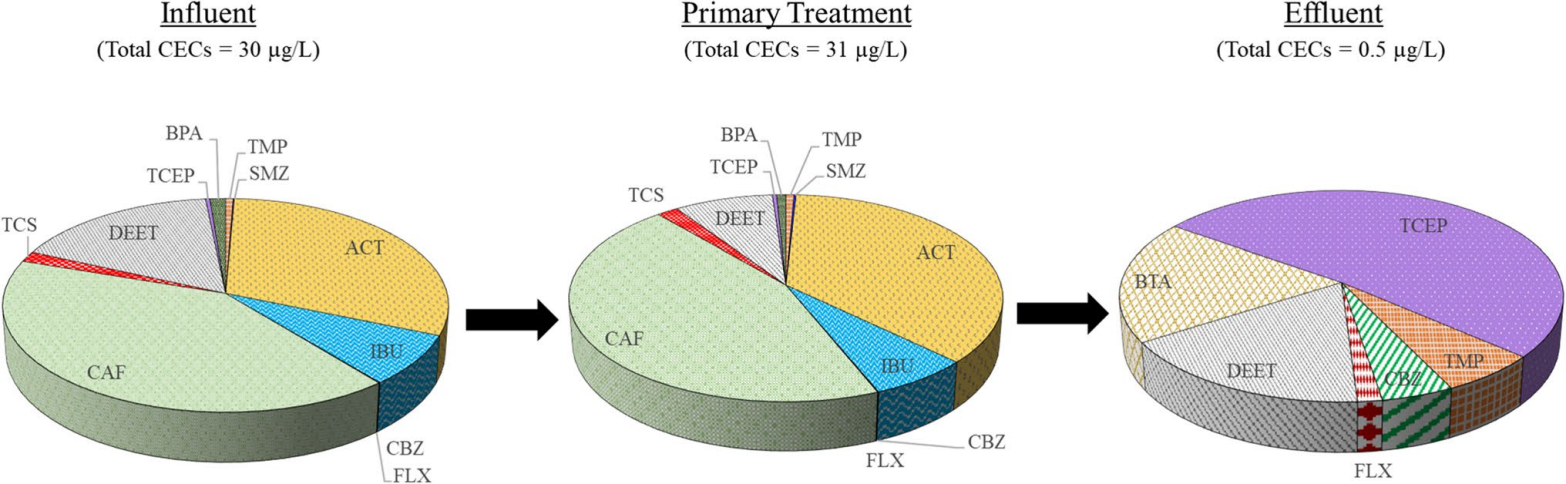
Distributions of quantified CECs

Figure 4 presents the total concentrations and relative proportion of CECs found in the WWTP at each sampling location. The total concentrations of CECs in the influent were around 30 µg/L. Fourteen target compounds were detected out of the twenty-two compounds investigated. The concentrations of CECs after primary treatments (in PT samples) slightly increased to 31 µg/L. Upon the secondary treatment (WSP), the concentrations of CECs significantly decreased to 0.5 µg/L. Despite their significantly reduced concentrations, many of the CECs were still present in the effluent, which would eventually pollute the receiving water and become a concern when the city’s proposed plan for reusing the water for drinking water supply is implemented. The concentrations of the target CECs and their concentrations in the Southeast Asia region, Asia, and the rest of the world are shown in Table 2 for comparative purposes.

**Table 2:** Concentrations of target CECs found in this study (mean ± SD, *n* = 2) and their range of concentrations from other studies in different geographical regions (ng/L)

CECs	This study			Southeast Asia			Asia			Other regions		
	Influent	PT	Effluent	Influent	Effluent	Ref	Influent	Effluent	Ref	Influent	Effluent	Ref
*Antibiotics*												
TMP	182 ± 13	211 ± 3	31 ± 0	4–313	2–369	(Rashid and Liu 2021; Shimizu et al. 2013; Yacob et al. 2017)	20–570	3.7–772	(Tran et al. 2018)	< LOQ–11,136	< LOQ–37,000	(Archer et al. 2018; Tran et al. 2018)
CLTR	n.d	n.d	n.d	< LOD–1854	1–637	(Kuroda et al. 2015; Shimizu et al. 2013; Tran et al. 2016)	26–1854	4.79–637.1	(Tran et al. 2018)	< LOQ–8000	0.3–7000	(Couto et al. 2019; Tran et al. 2018)
SMX	Detected*	Detected*	n.d	3–2600	< LOD–1066	(Kuroda et al. 2015; Menon et al. 2020; Shimizu et al. 2013; Tewari et al. 2013)	3–3930	< LOQ–1147	(Ben et al. 2018; Couto et al. 2019; Tran et al. 2018)	< LOQ–11,555	< LOQ–1800	(Tran et al. 2018)
SMZ	25 ± 3	54 ± 0	< LOD	1–1854	< LOD–260	(Shimizu et al. 2013; Tewari et al. 2013; Tran et al. 2016)	< LOQ–1814	< LOQ–260.8	(Ben et al. 2018; Tran et al. 2018)	< LOQ–680	< LOQ–363	(Tran et al. 2018)
*NSAIDs*												
ACT	9111 ± 137	11,273 ± 149	< LOD	< LOD–40,165	< LOD–9299	(Menon et al. 2020; Yacob et al. 2017)	67–147,700	< LOQ–2568	(Tran et al. 2018)	< LOQ–500,000	< LOQ–62,000	(Tran et al. 2018)
NPX	n.d	n.d	n.d	< LOD–7762	< LOD–2415	(Kuroda et al. 2015; Tran and Gin 2017)	< LOQ–7762	< LOQ–159	(Tran et al. 2018)	< LOQ–611,000	< LOQ–33,900	(Tran et al. 2018)
DFC	n.d	n.d	n.d	10–2107	4–650	(Kuroda et al. 2015; Tan et al. 2015; Tewari et al. 2013)	13–445	< LOQ–69.2	(Tran et al. 2018)	< LOQ–4869	< LOQ–5164	(Tran et al. 2018)
IBU	2315 ± 17	2069 ± 47	< LOD	< LOD–55,975	< LOQ–1135	(Kuroda et al. 2015; Tran and Gin 2017; Yacob et al. 2017)	34.8–55,975	< LOQ–1890	(Saidulu et al. 2021; Tran et al. 2018)	< LOQ–83,500	< LOQ–24,600	(Saidulu et al. 2021; Tran et al. 2018)
*Antimicrobial*												
TCS	470 ± 45	559 ± 35	< LOD	< LOQ–743	< LOQ–141	(Kuroda et al. 2015; Shimizu et al. 2013; Tran et al. 2016)	1.3–2500	5–263.9	(Tran et al. 2018)	< LOQ–128,000	< LOQ–430	(Gani et al. 2021; Tran et al. 2018)
*Beta-blockers*												
ATL	n.d	n.d	n.d	58–4602	< LOD–355	(Kuroda et al. 2015; Tan et al. 2015; Tran and Gin 2017)	< LOQ–294,700	< LOQ–1500	(Ben et al. 2018; Subedi et al. 2017; Tran et al. 2018)	< LOQ–33,106	< LOQ–14,200	(Archer et al. 2018; Couto et al. 2019; Tran et al. 2018)
MPL	n.d	n.d	n.d	22–959	17–189	(Al-Qaim et al. 2018; Tan et al. 2015)	< LOQ–79,500	< LOQ–335	(Ben et al. 2018; Tran et al. 2018)	< LOQ–4148	< LOQ–5762	(Tran et al. 2018)
*Anticonvulsant/antidepressants*												
CBZ	12 ± 1	12 ± 0	23 ± 0	< LOQ–339	< LOQ–336	(Tran and Gin 2017; Yacob et al. 2017)	< LOQ–18,500	< LOQ–900	(Ben et al. 2018; Tran et al. 2018)	< LOQ–3110	< LOD–4596	(Loos et al. 2013; Tran et al. 2018)
FLX	10 ± 0	8 ± 1	8 ± 0	NA	NA	–	< LOD–1310	< LOD–30	(Mole and Brooks 2019)	< LOD–3465	< LOD–2700	(Mole and Brooks 2019)
*Additives*												
BTA	< LOQ	< LOQ	92 ± 12	NA	NA	–	34.5–204	10.8–553	(Shi et al. 2019)	4800–75,000	14–100,000	(Shi et al. 2019)
*Plasticizers*												
BPA	378 ± 18	245 ± 26	< LOQ	128–6969	57–981	(Pookpoosa et al. 2015; Tran and Gin 2017)	55.6–5850	< LOQ–623	(Ben et al. 2018; Hu et al. 2019; Tran et al. 2018)	< LOQ–6230	2–1840	(Saidulu et al. 2021; Tran et al. 2018)
*Stimulants*												
CAF	12,220 ± 54	13,928 ± 27	< LOQ	3340–42,407	< LOQ–2940	(Al-Qaim et al. 2018; Li et al. 2012; Tran and Gin 2017)	759–61,000	13–51,700	(Huang et al. 2020; Subedi et al. 2017; Tran et al. 2018)	102–113,200	< LOQ–37,200	(Tran et al. 2018)
*Insect repellents*												
DEET	4968 ± 118	2592 ± 49	82 ± 0	93–2410	< LOQ–860	(Nguyen et al. 2018; Tan et al. 2015; Tran et al. 2018)	124–2341.9	21.6–324.8	(Huang et al. 2020; Tran et al. 2018)	< LOQ–42,334	< LOQ–1663	(Loos et al. 2013; Tran et al. 2018)
*Herbicides*												
ATZ	n.d	n.d	n.d	NA	NA	–	2	10–50	(Huang et al. 2020; Saidulu et al. 2021)	1–1650	4.2–36	(de Oliveira et al. 2020; Loos et al. 2013; Saidulu et al. 2021)
*Flame retardants*												
TCEP	101 ± 3	109 ± 3	251 ± 3	NA	NA	–	40–438	20–2620	(Huang et al. 2020; Kim et al. 2007; Zeng et al. 2015)	70–3000	71–6000	(Loos et al. 2013; Wang et al. 2020a, b; Xu et al. 2021)
*Antidiabetics*												
METF	Detected*	Detected*	n.d	NA	NA	–	2420–53,600	15–610	(Ambrosio-Albuquerque et al. 2021)	< LOQ–142,000	< LOQ–325,000	(Ambrosio-Albuquerque et al. 2021)
*Steroid hormones*												
E1	n.d	n.d	n.d	< LOQ–4299	< LOQ–369	(Rashid and Liu 2021; Tan et al. 2015; Tran et al. 2018)	< LOQ–241	< LOQ–51.2	(Ben et al. 2018; Saidulu et al. 2021; Tran et al. 2018)	2–670	< LOQ–95	(Couto et al. 2019; Kapelewska et al. 2018; Tran et al. 2018)
*DBPs*												
NDMA	n.d	n.d	n.d	NA	NA	–		16.1–247.9	(Li et al. 2019)		< LOD–537	(Jasemizad et al. 2021)

*Not quantified due to low SPE recoveries

*PT* post primary treatment, *NA* literature not available, *n.d.* not detected.

#### Occurrence of antibiotics

Three out of four antibiotics investigated were detected in influent, i.e., TMP (~ 182 ng/L), SMX (> LOD), and SMZ (25 ng/L). These antibiotics were reported to be present predominantly in sewage and waterways in tropical Asian countries (Shimizu et al. 2013). Shimizu et al. (2013) also identified these CECs in the nearby main river in Jakarta, Ciliwung River, at the average concentrations of 282 ng/L (SMX), 59 ng/L (TMP), and 23 ng/L (SMZ). Due to the lack of CECs’ occurrence data in the Citarum River, their level of occurrences in the effluent could not be directly compared with those possibly found in the receiving water body. However, our findings indicated that their concentrations in the effluent (31 ng/L for TMP and < LOD for SMZ) were on the lower side of their range of concentrations in the Ciliwung River (2–141 ng/L for TMP and 0–81 ng/L for SMZ) (Shimizu et al. 2013). Other potential sources of antibiotics discharged into surface water include direct wastewater discharge from hospitals and pharmaceutical industries, aquaculture and livestock production, untreated municipal sewage, and landfill leachate as shown in Fig. 1. To date, no study has investigated the antibiotic occurrences in hospital and pharmaceutical wastewater effluent in Indonesia.

TMP and SMZ found in this study were within their levels in Asian wastewater. The simultaneous occurrence of SMX and TMP has been reported since both antibiotics are often prescribed together (Kairigo et al. 2020). The distribution of pharmaceuticals in wastewater is notably influenced by their consumption patterns in the country (Tran et al. 2018). Unfortunately, acquiring an updated and reliable data on antibiotic consumption in Indonesia is challenging due to the inadequate documentation and data management systems, the lack of data transparency and information sharing, and the prevalence of unprescribed antibiotics use (Parathon et al. 2017). Pradipta et al. (2015) listed the total consumption of the top fourteen antibiotics in 61 community health centers in Bandung City from 2008 to 2010. They recorded the three most consumed antibiotics were amoxicillin, SMX, and TMP, which was in accordance with our detection of SMX and TMP. High rates of *Escherichia coli* isolates resistant to TMP-SMX (56%), second after ampicillin (73%), were discovered in two hospital wastewater discharges of Java Island (Parathon et al. 2017), indicating these CECs were among the two considerably consumed antibiotics in the country as well. SMZ has been predominantly found in livestock wastewater and thereby is often used as an indicator of the presence of livestock wastewater in wastewater influent (Shimizu et al. 2013). The occurrence of SMZ found in this study indicated that the wastewater influent was composed of a certain proportion of livestock wastewater. Bandung’s climatic conditions are deemed to be suitable for the farming and agriculture industry (Putro et al. 2021). They recorded around 12,000 traditional dairy farmers spread within the city in 2019, which could contribute to the WWTP influent.

CLTR (macrolide antibiotic) was not detected. The compound was not present on the list of the top fourteen most consumed antibiotics (Pradipta et al. 2015), hinting its low consumption in the city. Wang et al. (2020a, b) identified that macrolides contained in wastewater in Asia were lower than their occurrence in North America and Europe. Shimizu et al. (2013) highlighted macrolides were less commonly prescribed in Asian countries considering their considerably greater cost than the sulfonamides, e.g., SMX and TMP.

Antibiotics in aquatic environments may result in the proliferation of antibiotic-resistant bacteria (ARB) and antibiotic-resistant genes (ARGs) (Chereau et al. 2017). Southeast Asian countries are at high risk of the spread of human ARB and ARGs (Chereau et al. 2017). However, studies on ARB and ARGs in aquatic environments in these countries are still scarce. Only one study has been conducted in Indonesia; the study reported a substantial increase in ARGs in rivers downstream of city areas in the Central Java Province (Muurinen et al. 2022). Future studies on ARB and ARGs in the region are therefore required.

#### Occurrence of NSAIDs and stimulants

ACT and IBU were detected in the influent out of four investigated NSAIDs at 9.1 µg/L and 2.3 µg/L, respectively. CAF (stimulant) was found in the influent at 12.2 µg/L. Overall, ACT, IBU, and CAF were among the five most prevalent CECs in the influent. Likewise, Kuroda et al. (2015) discovered ACT and CAF as the two most ubiquitous PPCPs in the municipal wastewater influent in Hanoi, Vietnam. The predominant occurrence of ACT (8.6 µg/L) and CAF (9.1 µg/L) was also evident in seven WWTPs’ influents in Bangkok, Thailand, at similar concentrations detected in this study (Li et al. 2012). Similarly, consistently high levels of ACT (3.2–40.2 µg/L) and CAF (24.4–26.5 µg/L) were detected in several WWTP influents and along a river in Johor Bahru, Malaysia (Yacob et al. 2017), and Selangor, Malaysia (Tan et al. 2015).

ACT is a globally popular pain killer which can be obtained over-the-counter and prescribed (Hidayati et al. 2021). The use of ACT in Indonesia, where self-medication is preferred, is notably extensive (Hidayati et al. 2021; Koagouw et al. 2021). The tablet of ACT (500 mg) has always been listed as the number 2 in the Bappenas top 50 pharmaceuticals list, indicating high consumption in the country (Bappenas 2019). Previous studies also reported the presence of ACT in other environmental matrices in Indonesia. The compound was detected in the Jakarta Bay at 420–610 ng/L (Koagouw et al. 2021) and along the Central Java coastline (up to 11.3 ng/L and detection frequency of > 80%) (Hidayati et al. 2021), which indicated its widespread consumption in Java Island.

Both IBU 400 mg and 200 mg tablets have also been consistently listed in the top 30 in the Bappenas top 50 pharmaceuticals list (Bappenas 2019), hinting a relatively high consumption in the country. IBU occurrence in 13 rivers flowing to Jakarta Bay at 0.03–2.9 µg/L has been reported (Dsikowitzky et al. 2016, 2018), which corroborates our considerably high detection of IBU. The concentration of IBU in the effluent decreased to lower than LOD. ACT and IBU as the most abundant NSAIDs were also reported in wastewater influents in Singapore (Tran and Gin 2017). The concentrations of ACT and IBU found in this study were on the lower side of the range of those found in other countries.

NPX and DFC were not detected in this study. NPX was not listed in the top 50 pharmaceutical consumption list, while sodium diclofenac 50 mg tablet was ranked down the list (Bappenas 2019), which pointed to its significantly lower consumption than ACT and IBU. The non-detected results for DFC in our study could be attributed to its lower consumption and its lower dose per tablet (50 mg) as compared to ACT (100–500 mg) and IBU (200–400 mg). However, the occurrence of DFC in rivers flowing to Jakarta Bay (0–70 ng/L) has been reported (Dsikowitzky et al. 2016).

The predominant ACT occurrence in our study validates its prevalent usage, globally. ACT has been known to be the most commonly used medicine worldwide (McCudden 2020). Using the wastewater-based epidemiology approach, Yan et al. (2021) studied the consumption of four NSAIDs, i.e., ACT, IBU, DFC, and NPX, in various countries. Based on the data from 160 WWTPs in 18 countries, including China, India, the USA, and many European countries, ACT was found to be the most consumed NSAIDs, with the population normalized mass loads at 29–17,430 mg/day/1000 inhabitants (Yan et al. 2021).

CAF was found to be the most abundant CEC in the influent, while its concentration was < LOQ in the effluent. Aside from its usage as a stimulant in medicine, CAF is used in different food products and drinks. Coffee contains the highest concentration of CAF than other forms of caffeine-containing drinks (Quadra et al. 2020). Quadra et al. (2020) noted that the trends of coffee consumption in Indonesia increased over time, and since the country has a lack of proper wastewater collection and treatment, Indonesia would experience further caffeine contamination in its environment. Not surprisingly, high concentrations of CAF (up to 8.9 µg/L) were reported in 17 out of 18 sampling points along the rivers flowing through Jakarta, which were similar to the highest concentrations reported in the US rivers (Dsikowitzky et al. 2016). High concentrations of CAF in waterways in Jakarta or other cities in Indonesia could result from contamination with untreated sewage and greywater as well. Most households in Indonesia discharge untreated greywater directly to the drain, which eventually flows into waterways (World Bank 2013).

#### Occurrence of anticonvulsant/antidepressants and beta-blockers

CBZ was detected in the influent and effluent at 12 ng/L and 23 ng/L, respectively, which was found to be considerably lower than its occurrences in other countries. However, Yacob et al. (2017) observed a close range of concentrations in wastewaters collected from sewage treatment plants in Johor, Malaysia, i.e., < LOQ–19 ng/L in the influent and < LOQ–125 ng/L in the effluent. Elevated concentrations of CBZ in the effluent wastewater were reported by a number of studies in Asia (Kuroda et al. 2015; Tran and Gin 2017; Yacob et al. 2017) and in other regions (Hoque et al. 2014; Li et al. 2013). Although CBZ was not reflected in the top 50 pharmaceuticals in the country (Bappenas 2019), it is widely known as a common medicine to treat epileptic patients and can be purchased over the counter. Rahajeng et al. (2018) explained around 36% of off-label use of CBZ, such as neuropathic pain, nociceptive pain, and other indications, in four hospitals in Yogyakarta, Indonesia, indicating its other possible common use in the country. No study has investigated the occurrence of CBZ in the receiving waters in Indonesia.

FLX, an antidepressant, was found in both influent (10 ng/L) and effluent (8 ng/L). Several studies noted that the medicine is used as the most frequently prescribed antidepressant (73–89%) in major psychiatric hospitals on Java Island (Puspitasari and Angeline 2019; Sentari 2020), which pointed to its frequent usage in the country. Studies investigating the occurrence of antidepressants or widely known as selective serotonin reuptake inhibitors, like FLX, have been predominantly conducted in Europe and North America (Mole and Brooks 2019). No study has been performed in the Southeast Asia region to date, and only very few studies were recorded in the rest of Asia (Hong Kong and China) (Mole and Brooks 2019). FLX concentrations detected in this study were within the range of those reported in China. Significantly higher concentrations of FLX were found outside Asia.

MPL and ATL, cardio beta-blockers, were not detected in this study. Both compounds have been identified as the two most ubiquitous beta-blockers, detected in WWTPs in India, the USA, and Europe (Ahmad et al. 2022). As shown in Table 1, ATL was significantly more abundant than MPL in wastewater globally. ATL was also recorded to have a substantially higher occurrence in Asian wastewater than in other regions such as Europe and America (Ahmad et al. 2021). Several reasons could explain the non-detection of ATL and MPL in our study. Firstly, consumption of beta-blockers in Indonesia is expected to be considerably low, considering none of the beta-blockers is listed in the Bappenas top 50 pharmaceuticals list (Bappenas 2019). Secondly, cardioselective beta-blockers such as ATL and MPL are still considered not widely available in Indonesia; hence, they are less prescribed (Rizki and Siswanto 2014) and are not available over-the-counter. Additionally, studies have reported the use of other beta-blockers, e.g., bisoprolol and propranolol, by the Indonesian population (Rahmawati et al. 2019; Sari 2020).

#### Occurrence of antimicrobials, additives, and plasticizers

TCS was detected in the influent at 470 ng/L, which was around the median of its concentrations in Southeast Asian wastewater reported values. The compound was not detected in the effluent. Previous studies have investigated the occurrence of TCS in rivers in Jakarta (0–141 ng/L) (Dsikowitzky et al. 2016, 2018; Shimizu et al. 2013). TCS has not been regulated in Indonesia.

Studies on BTA occurrence in Asian wastewaters have been performed only in India and China (Shi et al. 2019). In our study, BTA was detected in the influent (< LOQ) and effluent (92 ng/L). Elevated concentrations of BTA in the effluent likely resulted from the breakdown of other complex molecules or the transformation of its conjugates (Shi et al. 2019). BTA is applied as an anticorrosion agent in many products, including metals, dishwashing detergents, and clothing textiles (Shi et al. 2019). A preliminary investigation by Brigden et al. (2013) observed the occurrence of BTA in wastewater effluent of textile industry in Bandung City, confirming the use of BTA in their production process, although the concentration was not reported. The washing of BTA-containing clothes was another potential source of BTA in domestic wastewater (Luongo et al. 2016), which could be the reason for BTA detection in wastewater of our study.

The influent concentration of BPA in the influent (378 ng/L) was close to the reported BPA concentrations (126–606 ng/L) in several WWTPs in Bangkok, Thailand (Pookpoosa et al. 2015). Rashid and Liu (2021) reported the occurrence of BPA in wastewater in Malaysia at up to 2.7 µg/L (influent) and 65 ng/L (effluent). Previous studies have reported the presence of BPA in rivers in Jakarta at 50–420 ng/L (Dsikowitzky et al. 2016, 2018).

The discharge of industrial wastewater and contamination of untreated domestic wastewater could potentially contribute to the high levels of CECs, including BPA, in surface waters (Kairigo et al. 2020). BPA was not detected in the Citarum River downstream of Bandung City (Kido et al. 2009). However, it is worth noting that the study was conducted in the year 2006, when BPA usage in the city might have been significantly lower than the current usage. BPA use in Indonesia increased by more than 450% from 2009 to 2013 (Perwita et al. 2015) and was projected to increase continually, given the absence of BPA regulations in food plastic containers, unlike in many developed countries (Mahamuni and Shrinithivihahshini 2017).

#### Occurrence of other CECs

DEET, insect repellent, was detected in the influent at ~ 5 µg/L, which was around twice its maximum reported concentrations in Southeast Asian and the rest of Asian wastewaters. Exceptionally high concentrations of DEET in rivers in Jakarta have been reported (0.02–35 µg/L), with maximum concentrations notably higher than DEET concentrations in any surface waters globally (Dsikowitzky et al. 2016, 2018). The use of insect repellent containing DEET in Indonesia is common, similar to other tropical countries (Merel and Snyder 2016). Once applied, DEET would be washed out during bathing or washing of hands and eventually end up in wastewater (Merel and Snyder 2016). DEET concentrations were significantly lower (82 ng/L) in the effluent due to the treatments used in the WWTP, which will be discussed in the section “Removal of CECs”.

Studies on TCEP occurrence in Asia have been conducted mainly in China and a few in South Korea (Xu et al. 2021). Overall, the concentrations of TCEP were significantly higher in North America, Europe, and Australia than those in Asia. TCEP concentrations reported in this study (101 ng/L in influent and 251 ng/L effluent) were within the range of its reported values in Asia. The increased concentrations of TCEP in treated wastewater have been justified (Liang and Liu 2016; Shi et al. 2016) due to its considerably high solubility and persistence in water (Pang et al. 2016; Zeng et al. 2015). TCEP was detected along rivers flowing to Jakarta Bay at 0.01–4.7 µg/L (Dsikowitzky et al. 2016, 2018). Although there is no literature specifying the use of TCEP by manufacturers in Indonesia, the detection of TCEP found in our study and surface waters in Jakarta implied the presence of TCEP-containing products in the country, causing its release into the wastewater and surface waters. TCEP is used in packaging and plastic bottles for various household products and has been found in laundry wastewater (Dsikowitzky et al. 2016), which could contribute to its occurrence in domestic wastewater.

ATZ, E1, and NDMA were not detected in our study. ATZ is not used widely in the country. Utami et al. (2020) surveyed the pesticides’ usage by farmers in Upper Citarum River Basin (UCRB) in Bandung City. They identified metsulfuron-methyl and paraquat dichloride as mainly used herbicides. Still, they reported that both herbicides were below LOD in the Citarum River. METF was detected in all samples but was not quantified due to its very low recovery. Considering the high prevalence of diabetes in Indonesia (ranked 7^th^ globally) (Pangribowo 2020), the metformin 500 tablet is listed in the Bappenas top 50 pharmaceuticals in the country (Bappenas 2019).

Studies around the world have detected low levels of E1 and other natural hormones in wastewaters (Camacho-Muñoz et al. 2012; Tran and Gin 2017), which could be due to (1) their much lower discharge concentrations than the synthetic hormones and/or (2) their rapid degradation in the wastewater collection system. The degradation of E1 by sunlight along the 4.5 km of open channel sewer in our study could contribute to non-detection as discovered by Caupos et al. (2011).

Likewise, photodegradation of NDMA by solar radiation under laboratory and environmentally relevant conditions was simulated by NDMA.

### Removal of CECs

**Fig. 5:**
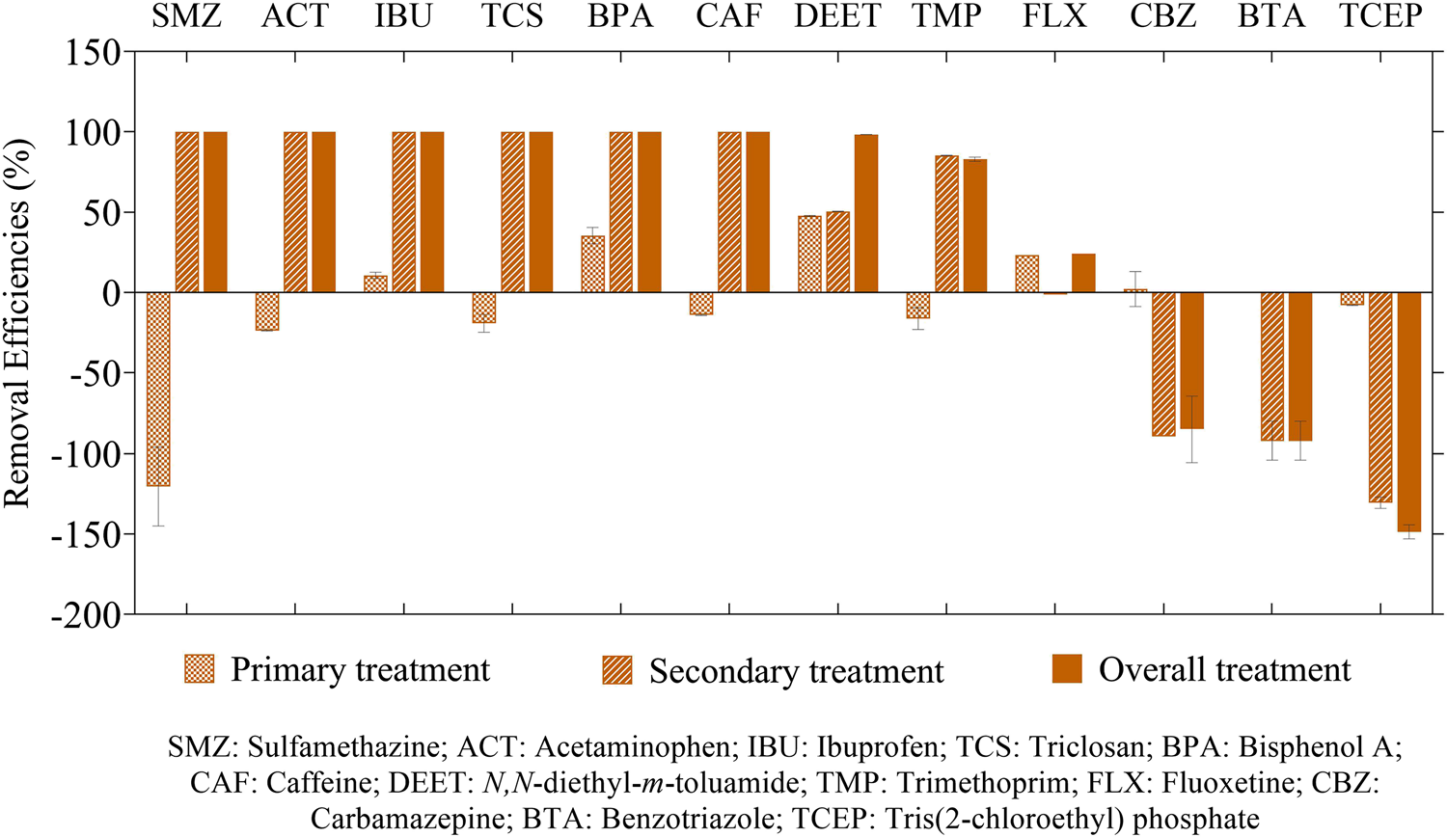
Removal efficiencies of quantified CECs by the WWTP. Negative removal efficiencies indicate CECs with higher concentrations after treatment

The removal efficiencies of the quantified CECs from the aqueous phase by primary, secondary, and overall treatments are shown in Fig. 5. The overall treatment yielded an efficient removal (~ 100%) for six CECs (SMZ, ACT, IBU, TCS, BPA, and CAF). Two compounds (DEET and TMP) were removed by 98% and 85%, respectively. Table S7 lists the removal efficiencies of the detected CECs by WSP systems from other studies. Although DEET removal by WSP has not been reported, its high removal efficiencies by conventional activated sludge (88–95%), Bardenpho treatment (96–97%), and aerated pond (> 95%) have been previously reported (Kumar et al. 2019; Nguyen et al. 2018; Tran and Gin 2017). In contrast, FLX was poorly removed (24%). Three compounds (CBZ, BTA, and TCEP) had higher concentrations in effluent than in influent. Overall, despite low TSS and organic content removal, the WSP was still efficient in the removal of the majority of detected CECs.

#### Removal by primary treatment

Primary treatment is generally ineffective in the removal of CECs (Baalbaki et al. 2017; Luo et al. 2014). The changes in CECs’ concentrations in primary sedimentation are mainly attributed to their sorption and desorption onto solid particles or sludge, which can be predicted from their water partition coefficients (log *K*_ow_) (Baalbaki et al. 2017). The adsorption constant, *K*_d_ (L/kg sewage sludge (SS)), also determines the extent of CECs’ adsorption onto sludge (Das et al. 2017). *K*_d_ is positively correlated to *K*_ow_ (Das et al. 2017). Adsorption is expected to be as follows: (1) ‘not significant’ for *K*_ow_ < 2.5, (2) ‘moderately significant’ for *K*_ow_ between 2.5 and 4, and (3) ‘highly significant’ for *K*_ow_ > 4 (Das et al. 2017). The values of log *K*_ow_ and log *K*_d_ of the detected CECs are listed in Table S7.

The concentrations of CECs with log *K*_ow_ < 2.5 (CAF, ACT, SMZ, TMP, TCEP, and CBZ) after primary treatment were either increased or insignificantly altered. CECs with log *K*_ow_ between 2.5 and 4 (BPA, IBU, and FLX) were moderately removed (11 to 36%). Interestingly, DEET (log *K*_ow_ 2.18) yielded 48% removal. DEET was often reported to have low removal efficiencies (< 20%) by primary treatment (Yang et al. 2017). However, a study conducted at WWTPs in China recorded around 40% removal of DEET by primary treatment (grit chamber and primary sedimentation) (Zhou et al. 2010). TCS with log *K*_ow_ of 4.76 showed a 19% higher effluent concentration. Although TCS removal by primary sedimentation has been recorded, from 42 to 94% (Yang et al. 2017), some studies also reported higher effluent concentrations (Behera et al. 2011; McAvoy et al. 2002). One of the possible causes of the observed significant variation for removal of CECs is the daily variation in their concentrations (Yang et al. 2017).

#### Removal by secondary treatment

In the secondary treatment, WSP, removal of CECs occurs through a number of mechanisms, i.e., biodegradation, sorption, photodegradation, and hydrolysis, with biodegradation as the primary removal pathway for most biodegradable compounds (Li et al. 2013). In warm temperatures (up to 37 °C), the growth of algae and microbial communities involved in biodegradation is optimum (Li et al. 2013), which benefits the WWTP in the removal of contaminants. The warm ambient temperatures in Bandung City played a key role in the high removal of the majority of detected CECs. When the study was conducted, the average daily temperatures were ~ 23 °C with maximum daily temperatures of 32 °C (BMKG 2019).

Previous studies suggested the use of physicochemical (log *K*_ow_ and log *K*_d_) and biological properties (*K*_bio_) of the CECs for predicting their removal mechanisms in biological treatment (Saidulu et al. 2021). *K*_bio_ refers to biodegradation rate constants (L/g SS/day) (Parida et al. 2021). They classified CECs based on the *K*_bio_ values: (1) highly biodegradable (*K*_bio_ > 10 L/g SS/day), (2) biodegradable (0.1 L/g SS/day < *K*_bio_ < 10 L/g SS/day), and (3) recalcitrant (*K*_bio_ < 0.1 L/g SS/day). CECs with log *K*_d_ less than 2.48 are usually not retained through sorption onto secondary sludge (Das et al. 2017; Xu et al. 2021).

The removal trends of the CECs detected in this study were consistent with their properties (Table S7). The CECs which were removed efficiently, such as SMZ, ACT, IBU, TCS, BPA, and CAF, have *K*_bio_ greater than 0.1 L/g SS/day (biodegradable). Compounds with high *K*_bio_ and low *K*_d_, such as ACT, IBU, CAF, and SMZ, were potentially removed primarily through biodegradation. Considering the high *K*_d_ of TCS and BPA, they were possibly removed through a combination of biodegradation and sorption. With TMP’s slightly higher *K*_d_, sorption likely was a primary mechanism in its removal, as also reported by Tran et al. (2016). TMP could be removed by photodegradation in WSP, albeit the removal varies significantly based on cloud cover, matrix effect, CECs, etc. (Ryan et al. 2011).

However, the removal of recalcitrant CECs by the WSP is limited (Kairigo et al. 2020). We found negligible removal of FLX after WSP treatment. Pomies et al. (2015) indicated FLX recalcitrancy (*K*_bio_ < 0.04 L/g SS/day) is caused by the three fluorine atoms on its structure. FLX removal by cyclic activated sludge has been reported at 23% (Blair et al. 2015). DEET was removed by 50% in the WSP. The moderate removal of this compound likely occurred through biodegradation, supported by its *K*_bio_ (0.1 < *K*_bio_ < 10) with low *K*_d_ (1.91), as shown in Table S7. Previous studies have confirmed the biodegradation of DEET by pure cultures of *Pseudomonas putida* and microorganisms from activated sludge WWTPs (Merel and Snyder 2016). The adapted microorganisms in the WSP biodegrading DEET could also be a possible reason for its higher removal than other CECs, as suggested by Merel and Snyder (2016).

The increased CBZ, TCEP, and BTA concentrations after biological treatment may occur due to several reasons such as (1) conjugation/deconjugation (Gewurtz et al. 2022), (2) recalcitrancy of the compounds, (3) ineffectiveness of the treatment, (4) desorption from the solid phase back to the aqueous phase, and (5) evaporation in a WSP system (Li et al. 2013). A recent study by Gewurtz et al. (2022) noted that conjugation and deconjugation impact the concentrations and fate of some pharmaceuticals and hormones during wastewater treatment and that their impact varies between analytes and WWTP process types. So, it may not be consistent for all analytes and for all treatments. Many studies found the negative removal of CBZ in WWTPs due to the abovementioned factors (Luo et al. 2014). Photodegradation could be an important mechanism for CBZ removal (Dai et al. 2012). However, its poor removal in this study indicated that sunlight exposure could not aid in removing the compound. CBZ was reported to be better removed in well-aerated treatments, such as oxidation ditch (Camacho-Muñoz et al. 2012) and activated sludge (Ying et al. 2009).

TCEP’s poor removal efficiencies were in agreement with its low log *K*_d_ (< 2.45), indicating that it was less susceptible to sorption. TCEP negligible removal by various WWTPs in different regions has been reported (Xu et al. 2021). The compound is also found to be removed less likely by biodegradation (Huang et al. 2020). BTA’s global occurrences in environmental water matrices were likely attributed to its hydrophilicity and low removal efficiencies by WWTPs (Shi et al. 2019). Despite its *K*_bio_ (0.22 L/g SS/day), suggesting that it is biodegradable, its removal in WWTPs shows significant variations (Gruchlik et al. 2018). For example, Matamoros et al. (2016) recorded BTA removal was around 45–55% by WSP and 72–77% by activated sludge. Voutsa et al. (2006) reported BTA removal by activated sludge at ten WWTPs which ranged from − 47 to 62%.

These results showed that the removal of CECs in WWTPs is a complex process which depends on many factors, such as the properties of CECs, their influent concentrations, wastewater matrix, and the WWTPs’ operational parameters (Das et al. 2017; Rodriguez-Narvaez et al. 2017). Negative removal or increased concentrations of CECs after treatment stages in WWTPs have been reported by a number of studies which have been attributed to several reasons. These include the transformation of human metabolites, CECs’ conjugate forming back their parent compounds, desorption from solid particles or sludge, and analytical uncertainty due to matrix interferences (Baalbaki et al. 2017; Luo et al. 2014).

### Aquatic risk assessment based on literature

The aquatic risk assessment was conducted by calculating the hazard quotients (HQs) using Eq. (1), as suggested by Matamoros et al. (2016). The aquatic risk was assessed for quantified CECs in the influent and effluent, considering the wastewater influent was contained in an open channel sewer, as mentioned earlier, and the effluent was discharged to a river.

1 $$\mathrm{HQ}=\frac{\mathrm{MEC}}{\mathrm{PNEC}}$$

MEC is the measured maximum concentration in the influent and effluent, and PNEC is the predicted no-effect concentration obtained from literature based on EC_50_ values (48 h) for *Daphnia magna* divided by 1000 (arbitrary safety factor). The results are shown in Table 3. In the influent, TCS had the highest HQ (1.32), indicating it posed a moderate risk (1 < HQ < 10) (Shi et al. 2016), followed by CAF, IBU, and DEET with HQ values between 0.1 and 1 (low risk). The cumulative HQ in the influent was 2.06 (moderate risk). The total HQ in the effluent was well below 0.1 (no risk associated).

**Table 3:** CECs’ maximum measured concentrations (MEC), EC_50_ values for *Daphnia magna* (48 h), PNEC, and hazard quotients (HQs)

CECs	Maximum MEC (ng/L)		EC50 (mg/L)	PNEC (µg/L)	HQ	
	Influent	Effluent			Influent	Effluent
*Antibiotics*						
TMP	195	31	149^a^	149	1.3E − 03	2.1E − 04
SMZ	28	< LOD	202^a^	202	1.4E − 04	
*NSAIDs*						
ACT	9248	< LOD	136^b^	136	6.8E − 02	0
IBU	2332	< LOD	9.02^c^	9.02	0.26	0
*Antimicrobial*						
TCS	515	< LOD	0.39^d^	0.39	1.32	0
*Anticonvulsant/antidepressants*						
CBZ	13	23	76.3^c^	76.3	1.7E − 04	3.0E − 04
FLX	10	8	6.4^e^	6.4	1.6E − 03	1.3E − 03
*Additives*						
BTA	< LOQ	104	107^f^	107	0	9.7E − 04
*Plasticizers*						
BPA	396	< LOQ	107^c^	107	3.7E − 03	0
*Stimulants*						
CAF	12,274	< LOQ	46^c^	46	0.27	0
*Insect repellents*						
DEET	5086	82	36.5^c^	36.5	0.14	2.2E − 03
*Flame retardants*						
TCEP	104	254	235^c^	235	4.4E − 04	1.1E − 03
Total					2.06	6.06E − 03

^a^De Liguoro et al. (2009)

^b^Jones et al. (2002).

^c^Matamoros et al. (2016).

^d^Orvos et al. (2002).

^e^Varano et al. (2017).

^f^Seeland et al. (2012)

## Conclusions

Fourteen out of twenty-two CECs were detected in the wastewater influent, with a total concentration of 29.8 ± 0.4 µg/L. Eight had remained in the effluent at a total concentration of 0.5 ± 0.0 µg/L. The occurrence of the CECs was found to be well correlated with their consumption and their known detection in surface waters in Indonesia. The concentrations of most CECs were within the range of those reported in Southeast Asia and the rest of Asia. DEET concentration was significantly higher than those recorded in wastewater in Asia. Biodegradable CECs were efficiently removed by the WSP even though COD removal was moderate only. In contrast, recalcitrant CECs with low adsorption capacities achieved poor removal in WSP. This study demonstrated the significance of having even conventional WWTP in removing or reducing the occurrence of CECs to minimize their contamination of the aquatic environment, particularly for countries with very limited coverage of centralized wastewater treatment like Indonesia. Although the WWTP achieved more than 98% removal of the detected CECs, certain CECs were still present in the effluent. It was found that the CECs in the influent resulted in moderate aquatic cumulative risk, with TCS posing a moderate risk and CAF, IBU, and DEET posing a low risk. No associated risk was predicted for the CECs in the effluent.

## Supplementary Information

Below is the link to the electronic supplementary material.

Supplementary file1 (DOCX 253 KB)

## Data Availability

Not applicable.

## Abbreviations

ACT: Acetaminophen
ARB: Antibiotic-resistant bacteria
ARGs: Antibiotic-resistant genes
ATL: Atenolol
ATZ: Atrazine
BOD: Biological oxygen demand
BPA: Bisphenol A
BTA: Benzotriazole
CAF: Caffeine
CBZ: Carbamazepine
CECs: Contaminants of emerging concerns
CLTR: Clarithromycin
COD: Chemical oxygen demand
DBPs: Disinfection byproducts
DEET: *N*-Diethyl-*meta*-toluamide
DFC: Diclofenac
E1: Estrone
FLX: Fluoxetine
HQs: Hazard quotients
IBU: Ibuprofen
LOD: Limit of detection
LOQ: Limit of quantification
MEC: Measured maximum concentration
METF: Metformin
MPL: Metoprolol
NDMA: *N*-Nitrosodimethylamine
NPX: Naproxen
NSAIDs: Non-steroidal anti-inflammatory drugs
PNEC: Predicted no-effect concentration
PPCPs: Pharmaceuticals and personal care products
SMX: Sulfamethoxazole
SMZ: Sulfamethazine
TCEP: Tris(2-chloroethyl) phosphate
TCS: Triclosan
TMP: Trimethoprim
TN: Total nitrogen
TOC: Total organic carbon
TP: Total phosphorus
TSS: Total suspended solids
WSP: Waste stabilization pond
WWTPs: Wastewater treatment plants
