# Correction to: Contaminants of emerging concerns (CECs) in a municipal wastewater treatment plant in Indonesia

**DOI:** 10.1007/s11356-022-24796-7

**Published:** 2022-12-23

**Authors:** Maryani Paramita Astuti, Suprihanto Notodarmojo, Cindy Rianti Priadi, Lokesh P. Padhye

**Affiliations:** 1 Department of Civil and Environmental Engineering, The University of Auckland, Auckland, New Zealand; 2 Environmental Engineering Study Program, Faculty of Engineering, President University, Cikarang, Indonesia; 3 Environmental Engineering Department, Faculty of Civil and Environmental Engineering, Bandung Institute of Technology (ITB), Bandung, Indonesia; 4 Environmental Engineering Study Program, Civil Engineering Department, Engineering Faculty, University of Indonesia (UI), Depok, Indonesia

**Correction to: Environmental Science and Pollution Research**

**https://doi.org/10.1007/s11356-022-23567-8**

The revised Figure 2 is shown in this paper.

**Fig. 2:**
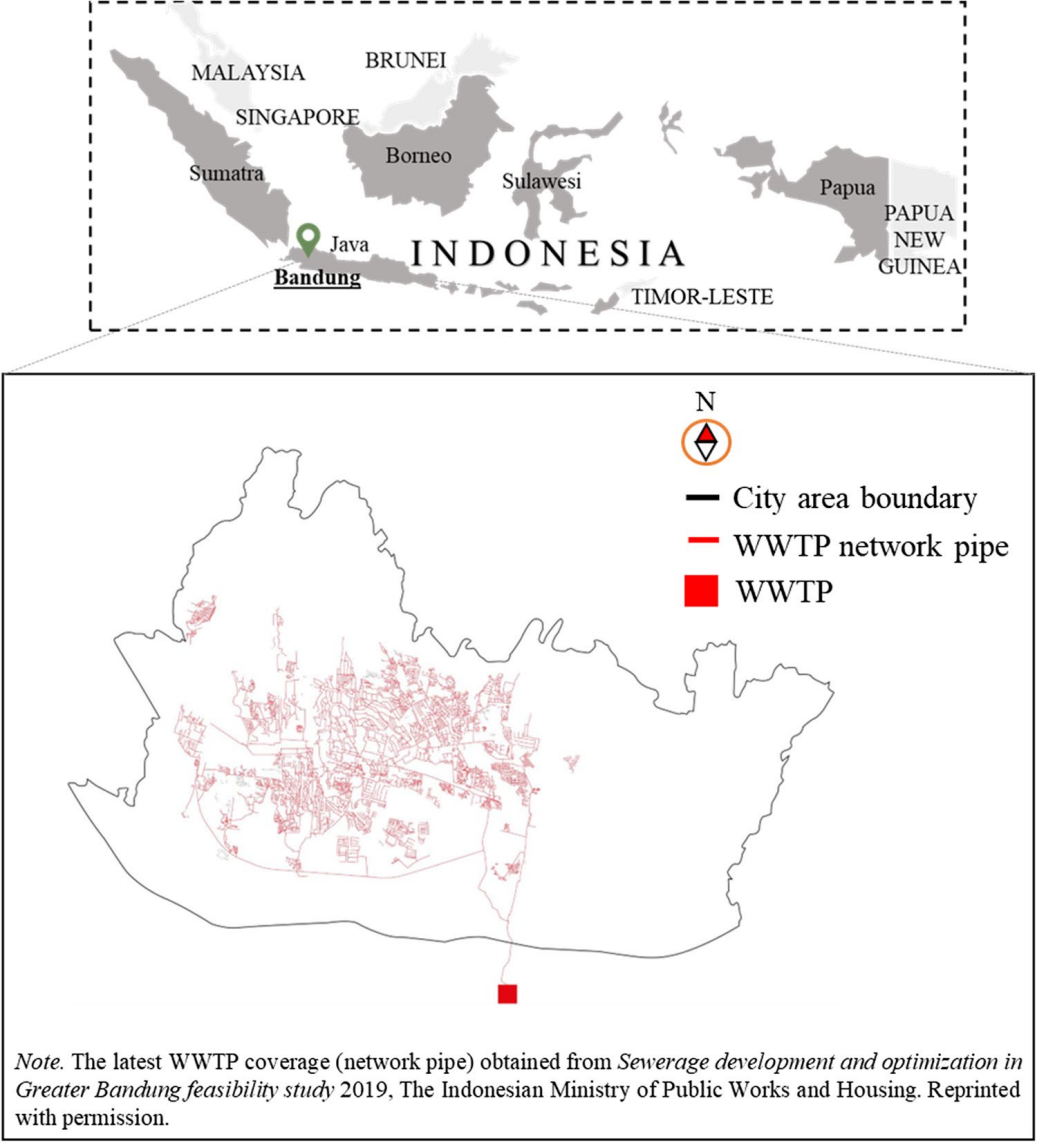
Location of Bandung City, the WWTP, and its coverage area

