# Supplementary material for: Contaminants of emerging concerns (CECs) in a municipal wastewater treatment plant in Indonesia
Source: Environ Sci Pollut Res Int. 2022 Oct 22;30(8):21512–32. doi: 10.1007/s11356-022-23567-8 (PMC9938049; doi:10.1007/s11356-022-23567-8)
Supplement: Supplementary file 1 — Supplementary file1 (DOCX 253 KB) [file 11356_2022_23567_MOESM1_ESM.docx]

Electronic Supplementary Materials for

**Contaminants of emerging concerns (CECs) in a municipal wastewater treatment plant in Indonesia**

***Maryani Paramita Astuti^a,b^, Suprihanto Notodarmojo^c^, Cindy Rianti Priadi^d^, and Lokesh P. Padhye^a^****

^a^Department of Civil and Environmental Engineering, The University of Auckland, Auckland, New Zealand.

^b^Environmental Engineering Study Program, Faculty of Engineering, President University, Cikarang, Indonesia.

^c^Environmental Engineering Department, Faculty of Civil and Environmental Engineering, Bandung Institute of Technology (ITB), Bandung, Indonesia.

^d^Environmental Engineering Study Program, Civil Engineering Department, Engineering Faculty, University of Indonesia (UI), Depok, Indonesia.

* Corresponding author. Tel: +64 09 923 2410; fax: +64 09 373 7462

E-mail address of the corresponding author: l.padhye@auckland.ac.nz

Abbreviations: Contaminants of emerging concerns (CECs); Pharmaceuticals and personal care products (PPCPs); Disinfection byproducts (DBPs); Wastewater treatment plants (WWTPs); Waste stabilization pond (WSP); Trimethoprim (TMP); Clarithromycin (CLTR); Sulfamethoxazole (SMX); Sulfamethazine (SMZ); Nonsteroidal anti-inflammatory (NSAIDs); Acetaminophen (ACT), Naproxen (NPX), Diclofenac (DFC); Ibuprofen (IBU); Triclosan (TCS); Atenolol (ATL); Metoprolol (MPL); Carbamazepine (CBZ); Fluoxetine (FLX); Benzotriazole (BTA); Bisphenol A (BPA); Caffeine (CAF); *N,N*-diethyl-*m*-toluamide (DEET); Atrazine (ATZ); Tris(2-chloroethyl) phosphate (TCEP); Metformin (METF); Estrone (E1), *N*-nitrosodimethylamine (NDMA).

**Tables (7)**

**Table S1.** The contaminants of emerging concerns (CECs) selected in the study

| **CECs** | **Abbreviation** | **Chemical Structure** |
| --- | --- | --- |
| ***Antibiotics*** |  |  |
| Trimethoprim | TMP |  |
| Clarithromycin | CLTR |  |
| Sulfamethoxazole | SMX |  |
| Sulfamethazine | SMZ |  |
| ***Nonsteroidal anti-inflammatory drugs (NSAIDs)*** |  |  |
| Acetaminophen | ACT |  |
| Naproxen | NPX |  |
| Diclofenac | DFC |  |
| Ibuprofen | IBU |  |
| ***Antimicrobial*** |  |  |
| Triclosan | TCS |  |
| ***Beta-blockers*** |  |  |
| Atenolol | ATL |  |
| Metoprolol | MPL |  |
| ***Anticonvulsants/Antidepressants*** |  |  |
| Carbamazepine | CBZ |  |
| Fluoxetine | FLX |  |
| ***Additives*** |  |  |
| Benzotriazole | BTA |  |
| ***Plasticizers*** |  |  |
| Bis-phenol A | BPA |  |
| ***Stimulants*** |  |  |
| Caffeine | CAF |  |
| ***Insect Repellents*** |  |  |
| *N,N*-diethyl-*m*-toluamide | DEET |  |
| ***Herbicides*** |  |  |
| Atrazine | ATZ |  |
| ***Flame Retardants*** |  |  |
| Tris(2-chloroethyl) phosphate | TCEP |  |
| ***Antidiabetics*** |  |  |
| Metformin | METF |  |
| ***Steroid Hormones*** |  |  |
| Estrone | E1 |  |
| ***Disinfection by-products (DBPs)*** |  |  |
| *N*-nitrosodimethylamine | NDMA |  |

**Table S2**. The design and operational parameters of the lagoon system in the WWTP

| Lagoon system | Parameters | | | |
| --- | --- | --- | --- | --- |
|  | Retention time (days) | Depth (m) | Surface area (hectares) | BOD removal (%) |
| Anaerobic pond | 2 | 4 | 4 | 60 |
| Facultative pond | 5-7 | 2 | 30 | 65 |
| Maturation pond | 3 | 1.5 | 32 | 40 |

**Table S3.** Mobile phase program

| Time (min) | Solvent B (%) |
| --- | --- |
| 0.01 | 10 |
| 10.0 | 30 |
| 30.0 | 30 |
| 50.0 | 90 |
| 52.0 | 10 |
| 60.0 | Stop |

**Table S4.** MS acquisition details of individual CECs

| **PPCPs** | **Molecular weight** | **Precursor ion** | **Product ion** | | **Collision Energy** | **Quantifier to qualifier ratio** | **Retention time** | **ESI polarity** |
| --- | --- | --- | --- | --- | --- | --- | --- | --- |
|  |  |  | **Quantifier ion** | **Qualifier ion** | **Quantifier, Qualifier** |  |  |  |
| Trimethoprim | 290.32 | 291 | 261.10 | 230.10 | -26, -24 | 70 | 16.600 | + |
| Clarithromycin | 733.93 | 748.50 | 158.20 | 590.40 | -28, -19 | 32.84 | 51.441 | + |
| Sulfamethoxazole | 253.279 | 254.20 | 156.00 | 92.10 | -15, -26 | 74.65 | 7.706 | + |
| Sulfamethazine | 278.33 | 279.00 | 186.00 | 124.00 | -17, -21 | 50.96 | 11.479 | + |
| Acetaminophen | 151.163 | 152.30 | 110.10 | 93.10 | -17, -24 | 31.58 | 7.339 | + |
| Naproxen | 230.26 | 229.30 | 185.20 | 170.20 | 7, 26 | 82.77 | 22.105 | - |
| Diclofenac | 296.148 | 294.10 | 250.10 | 214.10 | 12, 20 | 4.17 | 41.362 | - |
| Ibuprofen | 206.29 | 205.40 | 161.20 | - | 9 | - | 41.452 | - |
| Atenolol | 266.336 | 267.30 | 145.10 | 190.10 | -26, -18 | 73.22 | 8.739 | + |
| Metoprolol | 267.364 | 268.30 | 116.20 | 74.20 | -19, -22 | 92.83 | 24.413 | + |
| Carbamazepine | 236.269 | 237.30 | 194.10 | 179.10 | -19, -33 | 11.51 | 39.203 | + |
| Fluoxetine | 309.33 | 310.15 | 44.15 | 148.10 | -12, -8 | 9.28 | 50.302 | + |
| Triclosan | 289.54 | 286.80 | 35.05 | - | 70 | - | 52.468 | - |
| Benzotriazole | 119.12 | 120.30 | 65.10 | 92.10 | -22, -21 | 14.94 | 13.585 | + |
| Bisphenol A | 228.29 | 227.40 | 212.10 | 133.10 | 18, 27 | 35.33 | 45.745 | - |
| Caffein | 194.19 | 195.20 | 138.10 | 110.10 | -20, -21 | 21.51 | 10.708 | + |
| TCEP | 286.65 | 287.00 | 224.95 | 125.00 | -13, -16 | 92.91 | 36.480 | + |
| Atrazine | 215.68 | 216.10 | 174.15 | 132.10 | -17, -22 | 22.11 | 41.878 | + |
| DEET | 191.274 | 192.30 | 119.10 | 91.10 | -17, -30 | 91.88 | 40.967 | + |
| Metformin | 129.164 | 130.25 | 71.10 | 60.05 | -22, -13 | 84.77 | 2.284 | + |
| Estrone | 270.366 | 269.35 | 159.15 | 145.05 | 35, 41 | 12.23 | 2.700 | - |

**Table S5.** LODs, LOQs, and average recoveries of target CECs

| CECs | LODs  (ng/L) | LOQs (ng/L) | Recoveries Inlet (%) | Recoveries Pre-Bio (%) | Recoveries Outlet (%) |
| --- | --- | --- | --- | --- | --- |
| ***Antibiotics*** |  |  |  |  |  |
| TMP | 4 | 12 | 75±2 | 64±2 | 77±3 |
| CLTR* | 2 | 7 | 9±0.4 | 16±1 | 32±1 |
| SMX* | 37 | 120 | 3±2 | 4±0.4 | 16±0 |
| SMZ | 3 | 9 | 36±3 | 22±2 | 59±3 |
| ***NSAIDs*** |  |  |  |  |  |
| ACT | 13 | 40 | 76±7 | 78±2 | 119±2 |
| NPX | 109 | 360 | 97±5 | 94±1 | 98±5 |
| DFC | 20 | 63 | 68±1 | 59±1 | 61±0.1 |
| IBU | 76 | 250 | 48±5 | 38±0.3 | 58±0.3 |
| ***Antimicrobial*** |  |  |  |  |  |
| TCS | 83 | 274 | 53±1 | 40±0.3 | 57±1 |
| ***Beta-blockers*** |  |  |  |  |  |
| MPL | 12 | 40 | 101±2 | 102±6 | 106±8 |
| ATL* | 61 | 200 | 8±0.5 | 25±0.1 | 9±0.1 |
| ***Anticonvulsants/***  ***Antidepressants*** |  |  |  |  |  |
| CBZ | 3 | 10 | 72±2 | 100±0.4 | 79±2 |
| FLX | 1 | 3 | 79±1 | 77±0.2 | 78±0.3 |
| ***Additives*** |  |  |  |  |  |
| BTA | 20 | 60 | 109±1 | 118±3 | 112±6 |
| ***Plasticizers*** |  |  |  |  |  |
| BPA | 55 | 180 | 66±8 | 67±1 | 61±5 |
| ***Stimulants*** |  |  |  |  |  |
| CAF | 25 | 80 | 92±3 | 86±0.4 | 101±4 |
| ***Insect Repellents*** |  |  |  |  |  |
| DEET | 3 | 10 | 48±3 | 98±5 | 97±1 |
| ***Herbicides*** |  |  |  |  |  |
| ATZ | 6 | 20 | 128±2 | 135±5 | 131±4 |
| ***Flame Retardants*** |  |  |  |  |  |
| TCEP | 25 | 80 | 110±3 | 105±5 | 104±2 |
| ***Antidiabetics*** |  |  |  |  |  |
| METF* | 6 | 20 | 4±0.1 | 3±0.1 | 1±0.1 |
| ***Steroid Hormones*** |  |  |  |  |  |
| E1 | 6 | 20 | 41±1 | 44±0 | 41±7 |
| ***DBPs*** |  |  |  |  |  |
| NDMA** | 5 | 15 | - | - | - |

*CECs with very low (<20%) SPE recoveries

**NDMA was only analyzed for its presence/absence.

**Table S6.** Typical composition of domestic wastewater

| Parameters | Category^a^ | | |
| --- | --- | --- | --- |
|  | Weak | Medium | Strong |
| TSS (mg/L) | 100 | 220 | 350 |
| BOD (mg/L) | 110 | 220 | 400 |
| COD (mg/L) | 250 | 500 | 1000 |
| TOC (mg/L) | 80 | 160 | 290 |
| TN (mg/L) | 20 | 40 | 85 |
| TP (mg/L) | 4 | 8 | 15 |
| N-NH_4_^+^ (mg/L) | 12 | 25 | 50 |

a. (Metcalf et al. 2003).

**Table S7**. Removal efficiencies of the detected CECs by various WSP systems with seasonal variation and their relevant properties

| **CECs** | **Removal efficiencies (%)** | **Season/ Temperature** | | **Ref** | | **Log K_ow_** | | **Log K_d_** | | **K_bio_ (L/gSS/d)** | |
| --- | --- | --- | --- | --- | --- | --- | --- | --- | --- | --- | --- |
| ***Antibiotics*** |  |  | |  | |  | |  | |  | |
| TMP | 97 | Warm | | (Li et al. 2013) | | 0.91^a^ | | 1.41^a^ | | 0.29‒5.04^b^ | |
|  | -163 | Cold | |  |  |  | |  | | ‒1.44‒0.24^c^ | |
|  | 70.8 | Warm | | (Hoque et al. 2014) | |  | |  | |  | |
|  | 14 | Cold | |  |  |  | |  | |  | |
|  | 98-99 | - | | (K'Oreje et al. 2018) | |  | |  | |  | |
|  | 80-90 | - | | (Kairigo et al. 2020) | |  | |  | |  | |
| SMX | 98.9 | Warm | | (Hoque et al. 2014) | | 0.89^a^ | | 1.04^a^ | | 0.24^c^ | |
|  | 69.5 | Cold | |  |  |  | |  | | 0.19‒0.2^d^ | |
|  | 100 | - | | (Camacho-Muñoz et al. 2012) | |  | |  | | 0.01<K_bio_<0.1^e^ | |
|  | 58-99.7 | - | | (K'Oreje et al. 2018) | |  | |  | |  | |
|  | 97 | - | | (Muriuki et al. 2020) | |  | |  | |  | |
|  | 20, 80 | - | | (Kairigo et al. 2020) | |  | |  | |  | |
| SMZ | 26-99 | - | | (K'Oreje et al. 2018) | | 0.89^a^ | | 1.30 ^a^ | | 0.57^f f^ | |
| ***NSAIDs*** |  |  | |  | |  | |  | |  | |
| ACT | 100 | Warm | | (Matamoros et al. 2016) | | 0.46^i g^ | | 1.92^c^ | | 58‒80;106‒240^g^ | |
|  | 98 | Cold | |  |  |  | |  | |  | |
|  | ~100 | - | | (K'Oreje et al. 2018) | |  | |  | |  | |
|  | 79 | - | | (Kumar and Kumar 2020) | |  | |  | |  | |
| IBU | 99 | Warm | | (Li et al. 2013) | | 3.5^a^ | | 0.85^a^ | | 1.96‒70^h^ | |
|  | 81 | Cold | | (Li et al. 2013) | |  | |  | |  | |
|  | 99 | Warm | | (Matamoros et al. 2016) | |  | |  | |  | |
|  | 100 | Cold | | (Matamoros et al. 2016) | |  | |  | |  | |
|  | 91.5 | Warm | | (Hoque et al. 2014) | |  | |  | |  | |
|  | 78.3 | Cold | | (Hoque et al. 2014) | |  | |  | |  | |
|  | 99 to ~100 | - | | (K'Oreje et al. 2018) | |  | |  | |  | |
|  | 77 | - | | (Ying et al. 2009) | |  | |  | |  | |
|  | 75 | - | | (Camacho-Muñoz et al. 2012) | |  | |  | |  | |
|  | 99 | - | | (Hijosa-Valsero et al. 2010) | |  | |  | |  | |
| ***Antimicrobial*** | |  | |  | |  | |  | |  | |
| TCS | 99 | Warm | | (Li et al. 2013) | | 4.76^a^ | | 3.59^a^ | | 1.2‒3.6^c^ | |
|  | 95 | Cold | | (Li et al. 2013) | |  | |  | |  | |
|  | 90 | Warm | | (Matamoros et al. 2016) | |  | |  | |  | |
|  | 88 | Cold | | (Matamoros et al. 2016) | |  | |  | |  | |
|  | 97.2 | Warm | | (Hoque et al. 2014) | |  | |  | |  | |
|  | 42.5 | Cold | | (Hoque et al. 2014) | |  | |  | |  | |
|  | 85 | - | | (Ying et al. 2009) | |  | |  | |  | |
| ***Anticonvulsant/Antidepressants*** | | |  | |  | |  | |  | |  |
| CBZ | -362 | Warm | | (Li et al. 2013) | | 2.45^a^ | | 1.95^a^ | | <0.008;<0.005; <0.06;<0.03<0.1^g^ | |
|  | -122 | Cold | | (Li et al. 2013) | |  | | 1.31‒1.83^h^ | | 0^c^ | |
|  | 29 | Warm | | (Matamoros et al. 2016) | |  | | 1^c^ | |  | |
|  | 73 | Cold | | (Matamoros et al. 2016) | |  | |  | |  | |
|  | -9.28 | Warm | | (Hoque et al. 2014) | |  | |  | |  | |
|  | -51.4 | Cold | | (Hoque et al. 2014) | |  | |  | |  | |
|  | 6 | - | | (Ying et al. 2009) | |  | |  | |  | |
|  | 12 | - | | (Camacho-Muñoz et al. 2012) | |  | |  | |  | |
|  | -79 to 89 | - | | (K'Oreje et al. 2018) | |  | |  | |  | |
| FLX | - | - | | - | | 4.05 ^a^ | | 0.699^a^ | | 0‒0.04^j^ | |
|  |  |  | |  | |  | | 0.7‒3^a^ | |  | |
| ***Additives*** |  |  | |  | |  | |  | |  | |
| BTA | 55 | Warm | | (Matamoros et al. 2016) | | 1.44^k^ | | 2.34^k^ | | 0.22^k^ | |
|  | 45 | Cold | | (Matamoros et al. 2016) | |  | |  | |  | |
| ***Plasticizers*** |  |  | |  | |  | |  | |  | |
| BPA | 89 | Cold | | (Matamoros et al. 2016) | | 3.32^a^ | | 2.28^k^ | | 0.48‒12.2^i^ | |
|  | 30 | Warm | | (Qiang et al. 2013) | |  | |  | |  | |
|  | 20 | Cold | | (Qiang et al. 2013) | |  | |  | |  | |
|  | ~100 | - | | (Froehner et al. 2011) | |  | |  | |  | |
| ***Stimulants*** |  |  | |  | |  | |  | |  | |
| CAF | 100 | Warm | | (Li et al. 2013) | | 0.01^l^ | | <1.4‒2.15^b^ | | >10^e^ | |
|  | 99 | Cold | | (Li et al. 2013) | |  | | <2.5^m^ | | 0.48‒156.24^b^ | |
|  | 97 | Warm | | (Matamoros et al. 2016) | |  | |  | |  | |
|  | 100 | Cold | | (Matamoros et al. 2016) | |  | |  | |  | |
|  | 98 | - | | (Ying et al. 2009) | |  | |  | |  | |
|  | 99.6 | - | | (Froehner et al. 2011) | |  | |  | |  | |
|  | 65 | - | | (Camacho-Muñoz et al. 2012) | |  | |  | |  | |
|  | 83 | - | | (Hijosa-Valsero et al. 2010) | |  | |  | |  | |
| ***Insect Repellents*** | |  | |  | |  | |  | |  | |
| DEET | - | - | | - | | 2.18^a^ | | 1.91^a^ | | 0.109^e^  0.3^p^ | |
| ***Flame Retardants*** | |  | |  | |  | |  | |  | |
| TCEP | 28 | Warm | | (Matamoros et al. 2016) | | 1.44^n^ | | <2.45^n^ | | - | |
|  | 14 | Cold | | (Matamoros et al. 2016) | |  | |  | |  | |
| ***Flame Retardants*** | |  | |  | |  | |  | |  | |
| METF | - | - | | - | | ‒4.9^o^ | | 1.59^o^ | | - | |

K_ow_, octanol-water partition coefficient; K_d_, adsorption constant onto sludge (L/kg); K_bio_, biodegradation rate constants (L/gSS/d); a. (Das et al. 2017); b. (Parida et al. 2021); c. (Blair et al. 2015); d. (Muriuki et al. 2020); e. (Junwon 2016); f. (Kim and Zoh 2016); g. (Kosma et al. 2014); h. (Carballa et al. 2008); i. (Saidulu et al. 2021); j. (Pomies et al. 2015); k. (Garcia et al. 2021); l. (Gonçalves et al. 2017); m. (Xu et al. 2021); n. (Xu et al. 2021); o. (Briones et al. 2016); p. (Helbling et al. 2010).

**References**

Blair, B., Nikolaus, A., Hedman, C., Klaper, R. and Grundl, T. (2015) Evaluating the degradation, sorption, and negative mass balances of pharmaceuticals and personal care products during wastewater treatment. Chemosphere 134, 395-401.

Briones, R.M., Sarmah, A.K. and Padhye, L.P. (2016) A global perspective on the use, occurrence, fate and effects of anti-diabetic drug metformin in natural and engineered ecosystems. Environmental Pollution 219, 1007-1020.

Camacho-Muñoz, D., Martín, J., Santos, J., Aparicio, I. and Alonso, E. (2012) Effectiveness of conventional and low-cost wastewater treatments in the removal of pharmaceutically active compounds. Water Air Soil Pollut 223(5), 2611-2621.

Carballa, M., Fink, G., Omil, F., Lema, J.M. and Ternes, T. (2008) Determination of the solid-water distribution coefficient (Kd) for pharmaceuticals, estrogens and musk fragrances in digested sludge. Water Res 42(1-2), 287-295.

Das, S., Ray, N.M., Wan, J., Khan, A., Chakraborty, T. and Ray, M.B. (2017) Physico-Chemical Wastewater Treatment and Resource Recovery. Farooq, R. and Ahmad, Z. (eds), pp. 75-107, InTech, Croatiza.

Froehner, S., Piccioni, W., Machado, K.S. and Aisse, M.M. (2011) Removal capacity of caffeine, hormones, and bisphenol by aerobic and anaerobic sewage treatment. Water Air Soil Pollut 216(1), 463-471.

Garcia, L., Leyva-Diaz, J.C., Diaz, E. and Ordonez, S. (2021) A review of the adsorption-biological hybrid processes for the abatement of emerging pollutants: Removal efficiencies, physicochemical analysis, and economic evaluation. Sci Total Environ 780, 146554.

Gonçalves, E.S., Rodrigues, S.V. and Silva-Filho, E.V.d. (2017) The use of caffeine as a chemical marker of domestic wastewater contamination in surface waters: seasonal and spatial variations in Teresópolis, Brazil. Revista Ambiente & Água 12, 192-202.

Helbling, D.E., Hollender, J., Kohler, H.-P.E. and Fenner, K. (2010) Structure-based interpretation of biotransformation pathways of amide-containing compounds in sludge-seeded bioreactors. Environ. Sci. Technol. 44(17), 6628-6635.

Hijosa-Valsero, M., Matamoros, V., Martín-Villacorta, J., Bécares, E. and Bayona, J.M. (2010) Assessment of full-scale natural systems for the removal of PPCPs from wastewater in small communities. Water Res 44(5), 1429-1439.

Hoque, M.E., Cloutier, F., Arcieri, C., McInnes, M., Sultana, T., Murray, C., Vanrolleghem, P.A. and Metcalfe, C.D. (2014) Removal of selected pharmaceuticals, personal care products and artificial sweetener in an aerated sewage lagoon. Sci Total Environ 487, 801-812.

Junwon, P. (2016) Removal Characteristics and Predictive Model of Pharmaceutical and Personal Care Products (PPCPs) in Membrane Bioreactor (MBR) Process., Kyoto University, Japan.

K'Oreje, K.O., Kandie, F.J., Vergeynst, L., Abira, M.A., Van Langenhove, H., Okoth, M. and Demeestere, K. (2018) Occurrence, fate and removal of pharmaceuticals, personal care products and pesticides in wastewater stabilization ponds and receiving rivers in the Nzoia Basin, Kenya. Sci Total Environ 637-638, 336-348.

Kairigo, P., Ngumba, E., Sundberg, L.R., Gachanja, A. and Tuhkanen, T. (2020) Occurrence of antibiotics and risk of antibiotic resistance evolution in selected Kenyan wastewaters, surface waters and sediments. Sci Total Environ 720, 137580.

Kim, M.-K. and Zoh, K.-D. (2016) Occurrence and removals of micropollutants in water environment. Environmental Engineering Research 21(4), 319-332.

Kosma, C.I., Lambropoulou, D.A. and Albanis, T.A. (2014) Investigation of PPCPs in wastewater treatment plants in Greece: occurrence, removal and environmental risk assessment. Science of the Total Environment 466, 421-438.

Kumar, R. and Kumar, P. (2020) Wastewater Stabilisation Ponds: Removal of Emerging Contaminants. Journal of Sustainable Development of Energy, Water and Environment Systems 8(2), 344-359.

Li, X., Zheng, W. and Kelly, W.R. (2013) Occurrence and removal of pharmaceutical and hormone contaminants in rural wastewater treatment lagoons. Sci Total Environ 445, 22-28.

Matamoros, V., Rodríguez, Y. and Albaigés, J. (2016) A comparative assessment of intensive and extensive wastewater treatment technologies for removing emerging contaminants in small communities. Water Res 88, 777-785.

Metcalf, L., Eddy, H.P. and Tchobanoglous, G. (2003) Wastewater engineering: treatment, disposal, and reuse, McGraw-Hill, New York.

Muriuki, C., Kairigo, P., Home, P., Ngumba, E., Raude, J., Gachanja, A. and Tuhkanen, T. (2020) Mass loading, distribution, and removal of antibiotics and antiretroviral drugs in selected wastewater treatment plants in Kenya. Sci Total Environ 743, 140655.

Parida, V.K., Saidulu, D., Majumder, A., Srivastava, A., Gupta, B. and Gupta, A.K. (2021) Emerging contaminants in wastewater: A critical review on occurrence, existing legislations, risk assessment, and sustainable treatment alternatives. J Environ Chem Eng 9(5).

Pomies, M., Choubert, J.M., Wisniewski, C., Miege, C., Budzinski, H. and Coquery, M. (2015) Lab-scale experimental strategy for determining micropollutant partition coefficient and biodegradation constants in activated sludge. Environ Sci Pollut Res Int 22(6), 4383-4395.

Qiang, Z., Dong, H., Zhu, B., Qu, J. and Nie, Y. (2013) A comparison of various rural wastewater treatment processes for the removal of endocrine-disrupting chemicals (EDCs). Chemosphere 92(8), 986-992.

Xu, G., Zhao, X., Zhao, S., Chen, C., Rogers, M.J., Ramaswamy, R. and He, J. (2021) Insights into the occurrence, fate, and impacts of halogenated flame retardants in municipal wastewater treatment plants. Environ Sci Technol 55(8), 4205-4226.

Ying, G.-G., Kookana, R.S. and Kolpin, D.W. (2009) Occurrence and removal of pharmaceutically active compounds in sewage treatment plants with different technologies. J Env Monit 11(8), 1498-1505.
